# Dynamic Interplay in Tumor Ecosystems: Communication between Hepatoma Cells and Fibroblasts

**DOI:** 10.3390/ijms241813996

**Published:** 2023-09-12

**Authors:** Gábor Petővári, Gábor Tóth, Lilla Turiák, Anna L. Kiss, Krisztina Pálóczi, Anna Sebestyén, Adrián Pesti, András Kiss, Kornélia Baghy, Katalin Dezső, Tibor Füle, Péter Tátrai, Ilona Kovalszky, Andrea Reszegi

**Affiliations:** 1 Department of Pathology and Experimental Cancer Research, Semmelweis University, Üllői út 26, H-1085 Budapest, Hungary; 2 MS Proteomics Research Group, Research Centre for Natural Sciences, Eötvös Loránd Research Network, Magyar Tudósok Körútja 2, H-1117 Budapest, Hungary; 3 Department of Human Morphology and Developmental Biology, Semmelweis University, Tűzoltó u. 58, H-1094 Budapest, Hungary; 4 Department of Genetics, Cell and Immunobiology, Semmelweis University, H-1085 Budapest, Hungary; 5 Department of Pathology, Forensic and Insurance Medicine, Semmelweis University, Üllői út 93, H-1091 Budapest, Hungary; 6 Thermo Fisher Scientific Inc., Váci út. 41-43, H-1134 Budapest, Hungary; 7 Charles River Laboratories Hungary, Irinyi József utca 4-20, H-1117 Budapest, Hungary; 8 Department of Pediatrics, College of Medicine, University of Florida, Gainesville, FL 32610, USA

**Keywords:** hepatoma cell, fibroblast, tumor, stroma, cell communication, invasion, extracellular vesicle, miRNA, metabolism

## Abstract

Tumors are intricate ecosystems where cancer cells and non-malignant stromal cells, including cancer-associated fibroblasts (CAFs), engage in complex communication. In this study, we investigated the interaction between poorly (HLE) and well-differentiated (HuH7) hepatoma cells and LX2 fibroblasts. We explored various communication channels, including soluble factors, metabolites, extracellular vesicles (EVs), and miRNAs. Co-culture with HLE cells induced LX2 to produce higher levels of laminin β1, type IV collagen, and CD44, with pronounced syndecan-1 shedding. Conversely, in HuH7/LX2 co-culture, fibronectin, thrombospondin-1, type IV collagen, and cell surface syndecan-1 were dominant matrix components. Integrins α6β4 and α6β1 were upregulated in HLE, while α5β1 and αVβ1 were increased in HuH7. HLE-stimulated LX2 produced excess MMP-2 and 9, whereas HuH7-stimulated LX2 produced excess MMP-1. LX2 activated MAPK and Wnt signaling in hepatoma cells, and conversely, hepatoma-derived EVs upregulated MAPK and Wnt in LX2 cells. LX2-derived EVs induced over tenfold upregulation of SPOCK1/testican-1 in hepatoma EV cargo. We also identified liver cancer-specific miRNAs in hepatoma EVs, with potential implications for early diagnosis. In summary, our study reveals tumor type-dependent communication between hepatoma cells and fibroblasts, shedding light on potential implications for tumor progression. However, the clinical relevance of liver cancer-specific miRNAs requires further investigation.

## 1. Introduction

Cancer is a complex organization, an ecosystem of tumor cells surrounded by stromal components. The stroma is built up by non-tumorous cells like fibroblasts, inflammatory cells, and blood vessels, all embedded in extracellular matrix [1,2]. In the last 15 years, it became evident that these stromal components are all but innocent bystanders in the development and progression of malignant disease [3]. They can produce cytokines and growth factors, secrete matrix proteins, and feed the tumor cells [4,5]. In the end, they collectively create a microenvironment that fosters the growth and invasion of tumor cells.

However, there seems to be no general pattern that characterizes this microenvironment in every tumor [2]. The stroma is highly variable and specific for each particular tumor type and largely influences the success of cancer treatment [6]. Hence, to improve the efficacy of cancer management we must unravel the secrets of the tumor microenvironment.

Carcinogenesis is a multistep process initiated by genetic or epigenetic injuries of genes implicated in cell cycle regulation. But as prospective cancer cells undergo a gradual progression, so does the host microenvironment [7]. Thus, when Hanahan’s classical concept of carcinogenesis underwent revision and mechanisms that do not implicate genetic alterations were added as new hallmarks [8], epigenetic alteration of the tumor microenvironment was one of them, along with the role of the microbiome and cellular senescence.

The cellular components of the tumor stroma include endothelial cells and pericytes of preexisting or neoangiogenic blood vessels as well as tumor-associated fibroblasts. Although the latter most frequently originate from normal fibroblasts, several other cell types such as stromal cells of the bone marrow [9], pericytes, and hepatic or pancreatic stellate cells can transform into cancer-associated fibroblasts (CAF). This implies that CAF constitutes a heterogeneous cell population with properties depending on the cellular origin and localization in the body [10]. It is still debated whether different CAFs are different types of cells or if they specialize to different functions as a result of their plasticity [11,12,13]. However, irrespective of their origin, they become active participants in the tumor stroma which contribute to tumor progression in a variety of ways. By secreting collagens, fibronectin, and hyaluronic acid they modulate the stiffness of the tumor matrix; by synthesizing TGF-beta they contribute to the epithelial-to-mesenchymal transition (EMT), facilitate the upregulation of SMAD, and activate cytokines such as CXCL12. Moreover, by providing lactate and other metabolites to proliferating tumor cells, they effectively regulate the intermediate metabolism of the cells [1,10].

The tumor stroma typically also contains representative cell types of the innate and adaptive immune system. Whereas the innate immune system tends to promote tumorigenesis via inflammation and angiogenesis [14], adaptive immunity is poised towards hampering tumor progression through the action of dendritic cells and T cells [15]. In the realm of innate immunity, there exists the potential for harnessing its capabilities in the fight against immune evasion [16].

This in vitro study focuses on the communication of two hepatoma cell lines with the immortalized LX2 fibroblast cells. One key inquiry pertains to the rationale guiding the selection of specific signaling pathways and proteins for investigation. This decision was made with the aim of shedding light on fundamental aspects of hepatocellular carcinoma (HCC) progression. The signaling pathways and proteins chosen are known to play crucial roles in regulating key processes such as cell proliferation, matrix remodeling, and intercellular communication in hepatocellular carcinoma. In this study, we aimed to explore the intricate communication channels between poorly (HLE) and well-differentiated (HuH7) hepatoma cells and LX2 fibroblasts, mimicking the tumor microenvironment in vitro. Through a comprehensive examination of secreted soluble factors, metabolites, extracellular vesicles (EVs), and miRNAs, we aim to elucidate the tumor type-dependent communication network that underlies HCC progression. These findings have the potential to inform therapeutic strategies and may open new avenues for early diagnosis and intervention.

## 2. Results

### 2.1. Morphology, Proliferation, and Invasion of HLE and HuH7 Hepatoma Cell Lines and LX2 Liver Fibroblast Cells

**Figure 1:**
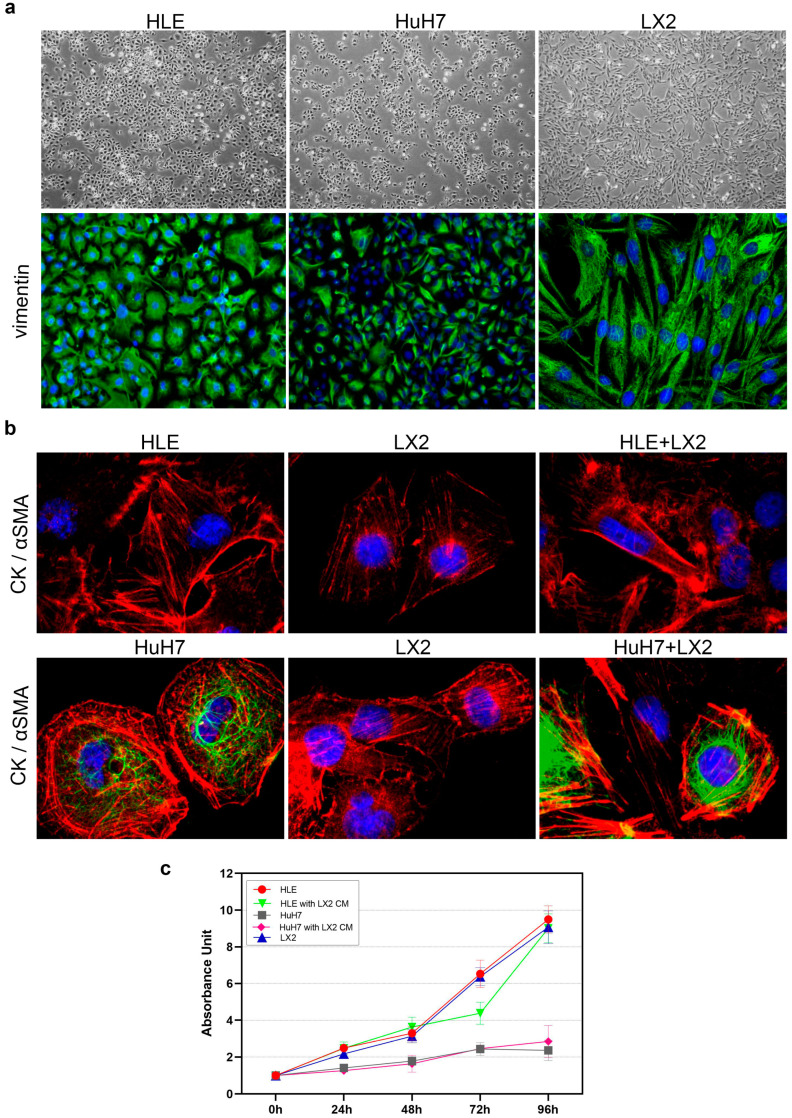
Characterization and proliferation rates of HLE, HuH7, and LX2. (**a**) Phase contrast micrographs and vimentin immunofluorescence of HLE, HuH7, and LX2 cells. HLE is a dedifferentiated, fast-growing hepatoma cell line whereas HuH7 is more differentiated and grows slower. All three cell lines were positive for vimentin. Vimentin (green) and DAPI (blue). Original magnification: 20× (phase contrast micrographs), 40× (HLE, HuH7 vimentin staining), and 63× (LX2 vimentin staining). (**b**) Double immunostaining of hepatoma cell lines kept in monoculture or in direct co-culture with LX2 cells. HLE cells did not express cytokeratin and both hepatoma cell lines were positive for αSMA. Cytokeratin (green), αSMA (red), and DAPI (blue). The original magnification in the confocal microscope was 63×. (**c**) Proliferation rates of HLE, HuH7, and LX2 cells and the effect of LX2-conditioned medium on HLE and HuH7 cell proliferation.

**Figure 2:**
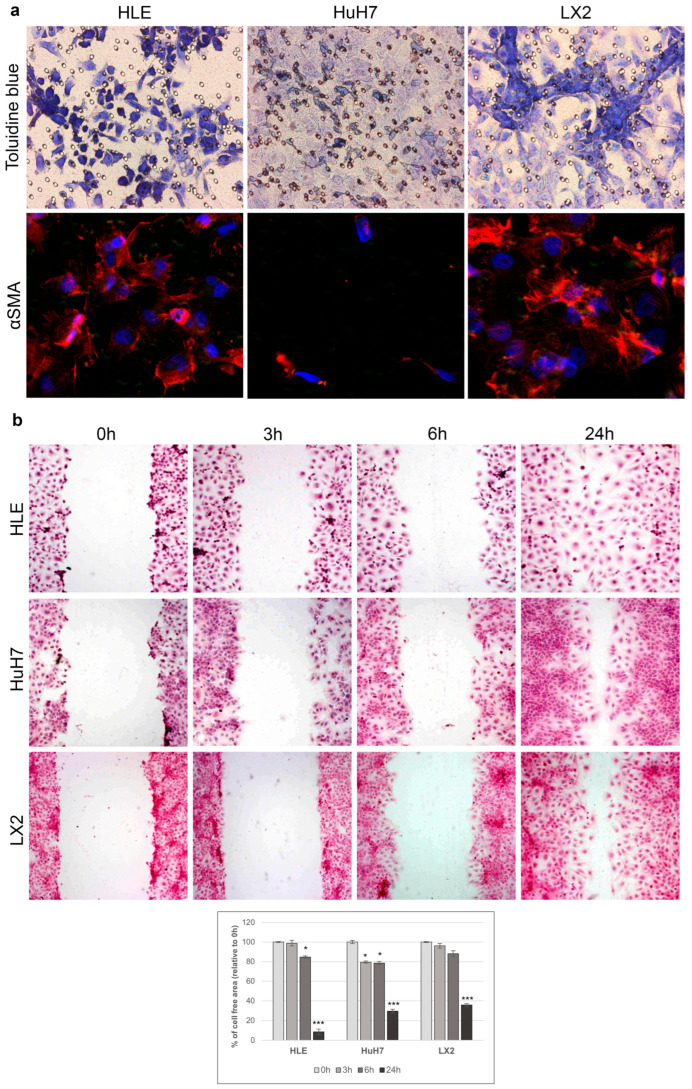
Migration and wound healing assays. (**a**) Migration in Boyden chamber: attracted by Matrigel, HLE, and LX2 cells, but not HuH7 cells, migrate through the pores of the membrane. Migrating cells were positive for αSMA. αSMA (red) and DAPI (blue). Original magnification: 20× (αSMA staining) and 40× (Toluidine blue staining). Wound healing assay and bar-graphs of the cell-free area over time: (**b**) microscope images presenting scratch area covered by migrating HLE, HuH7, and LX2 cells. Wound closure was evaluated by measuring the remaining cell-free area and expressed as a percentage of the initial (0 h) cell-free area. Quantification was performed by ImageJ analysis. HLE cells covered the scraped area within 24 h while wound closure of HuH7 and LX2 was still incomplete at this time point. The results of three independent experiments are expressed as mean ± SD of the percentage of the cell-free area. The original magnification of the images was 10×. * *p* < 0.05 and *** *p* < 0.001.

Characterization of the two hepatoma cell lines revealed that HLE consists of fast-growing dedifferentiated cells, in contrast with HuH7 cells that are more differentiated and grow slower (Figure 1 and Figure 2). Both hepatoma cell lines expressed vimentin and α-smooth muscle actin (α SMA) and HLE was negative for cytokeratin (Figure 1a,b).

The proliferation rate of HLE and LX2 cells was high, with the number of cells increasing more than eightfold within four days (Figure 1c); as a contrast, HuH7 cells only doubled within the same time interval and displayed modest proliferation (Figure 1c). The conditioned medium of LX2 cells did not further promote the proliferation of HLE cells (Figure 1c), whereas compared to control HuH7 cells that started to decline after 72 h, HuH7 cells growing in the LX2 medium continued to proliferate until the end of the experiment. However, it is important to note that this difference was not statistically significant (Figure 1c).

HLE and LX2 cells, but not HuH7, were able to migrate through the Boyden chamber when attracted by Matrigel (Figure 2a). Although all three cell lines were active in the wound healing assay, only HLE closed the wound within 24 h. LX2 immortalized liver fibroblasts retained their fibroblast phenotype (Figure 2b).

### 2.2. Crosstalk between Hepatoma Cells and LX2 via Soluble Mediators Mutually Modulates Signal Transduction and Downregulates Cyclin-Dependent Kinase Inhibitor p21

**Figure 3:**
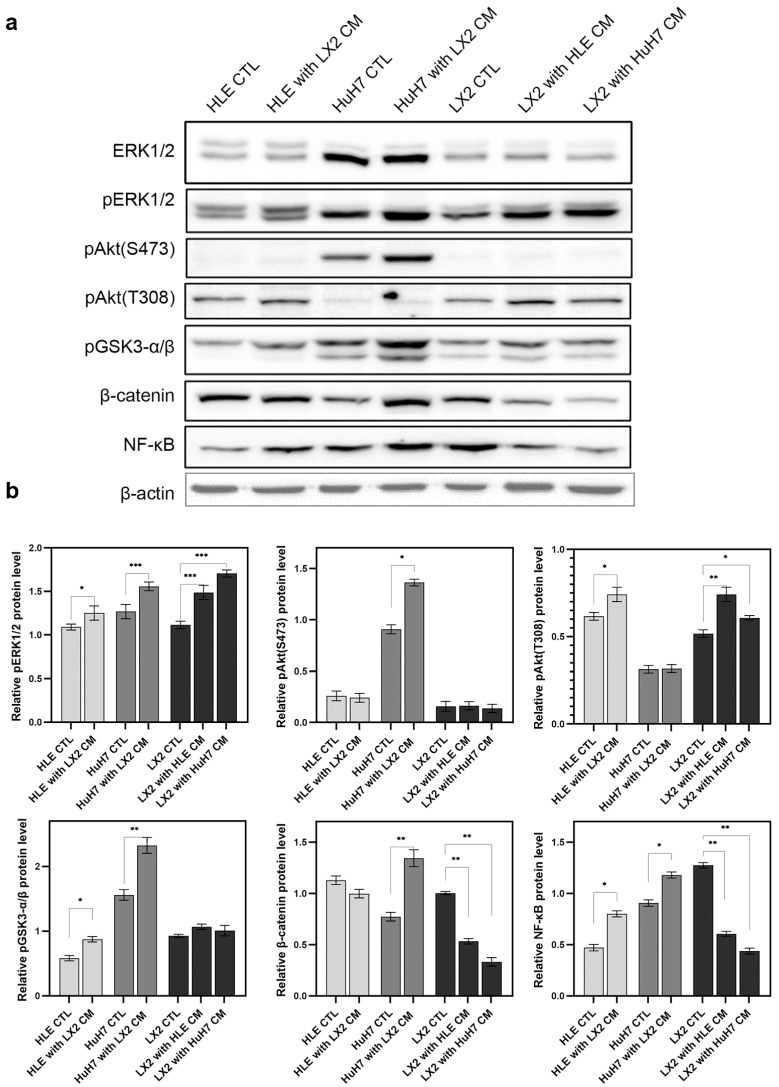
Reciprocal effects of the conditioned media of hepatoma cells and LX2 fibroblasts on cell signaling. Conditioned medium of LX2 activated the Akt/mTOR, MAPK, and NF-κB pathways and inhibited GSK3-α/β in both hepatoma cell lines. In the case of β-catenin, it increased in HuH7 cells and decreased in HLE cells in response to LX2-conditioned medium. Conditioned media of hepatoma cell lines activated ERK1/2 and Akt(T308) but downregulated β-catenin and NF-κB in LX2 cells. (**a**) Immunoblots and (**b**) corresponding densitometry graphs. β-actin housekeeping protein was used as a reference for protein loading and relative activity levels were compared to respective controls. Data points represent mean ± SD, n = 3; * *p* < 0.05; ** *p* < 0.01; and *** *p* < 0.001.

**Figure 4:**
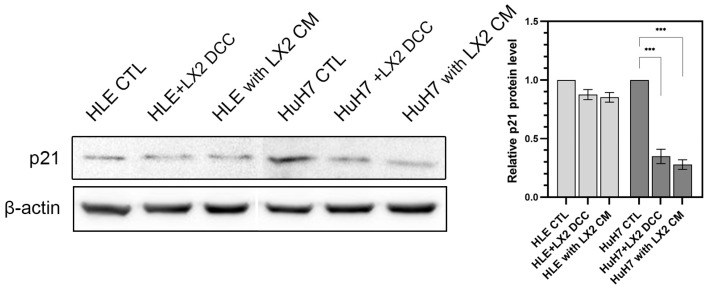
LX2 direct co-culture and conditioned medium downregulated the expression of cyclin-dependent kinase inhibitor p21 in HuH7 but only marginally in HLE where p21 was already suppressed in the control condition. Immunoblots (**left**) and corresponding densitometry graphs (**right**). β-actin housekeeping protein was used as a reference for protein loading and relative activity levels were compared to respective controls. Data points represent mean ± SD, n = 3; *** *p* < 0.001.

An undiluted conditioned medium of LX2 cells was applied to HLE and HuH7 cells, and conversely, the conditioned media of hepatoma cells was applied to LX2. After 2 days of treatment, the effect on the expression of regulatory proteins was assessed (Figure 3). Control HLE and HuH7 cell lines were grown in DMEM containing 10% (*v*/*v*) fetal calf serum. In both HLE and HuH7 cells, the LX2-conditioned medium increased activating phosphorylation of ERK1/2, Akt, and NF-κB as well as inhibitory phosphorylation of GSK3-α/β. In the case of β-catenin, it increased in HuH7 cells and decreased in HLE cells in response to the LX2-conditioned medium. The latter change was accompanied by the activation of β-catenin in HuH7 but not in HLE. In the reciprocal setup, the conditioned media of hepatoma cell lines activated ERK1/2, Akt(T308), and GSK3-α/β but downregulated β-catenin and NF-κB. LX2-conditioned medium suppressed the expression of the CDK inhibitor p21 in both hepatoma cell lines (Figure 4), albeit only marginally in the more aggressive HLE cells where baseline expression of p21 was low to start with.

### 2.3. Contact Co-Culture of Hepatomas and Fibroblasts Alters the Expression of ECM Proteins

**Figure 5:**
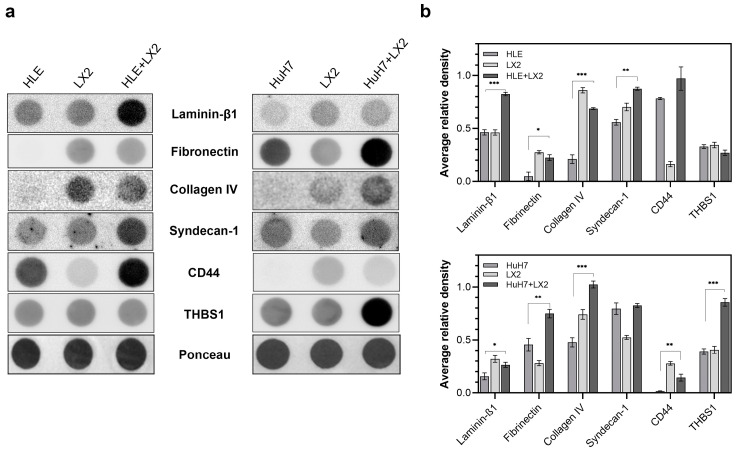
Secretion of matrix proteins into the culture media by single cultures and co-cultures of HLE, HuH7, and LX2. (**a**) Immunoblots and (**b**) corresponding densitometry graphs. Ponceau S staining was used as a reference for protein loading. Data points represent mean ± SD, n = 3; * *p* < 0.05; ** *p* < 0.01; and *** *p* < 0.001.

Hepatomas and LX2 cells were grown alone or in contact co-culture and ECM proteins were quantified in the media of single cultures and co-cultures (Figure 5). HLE alone secreted a modest amount of laminin-β1 and it facilitated laminin secretion of LX2 cells when grown in co-culture, which resulted in high amounts of laminin in the co-culture medium. On the other hand, isolated HuH7 cells secreted hardly any laminin-β1; thus, laminin in the co-culture medium was likely derived from LX2 cells.

HLE in isolation did not secrete fibronectin; thus, all fibronectin in the co-culture medium was likely secreted by LX2 cells. HuH7 cells, as a contrast, displayed autonomous secretion of fibronectin, contributing to the high total amount of fibronectin jointly produced by HuH7 and LX2 in the co-culture. HLE, but not HuH7, secreted a considerable amount of CD44 into the culture medium, which increased further in the medium of co-culture. Only low amounts of thrombospondin were secreted by any of the cell cultures alone but the same amount was abundant in the HuH7/LX2 co-culture medium. LX2, but neither HLE nor HuH7, secreted type IV collagen, and all cell cultures shed syndecan-1 into the culture media.

### 2.4. Expression of Matrix Metalloproteases (MMPs) Is Enhanced by Co-Culturing Hepatoma Cells with LX2 Fibroblasts

**Figure 6:**
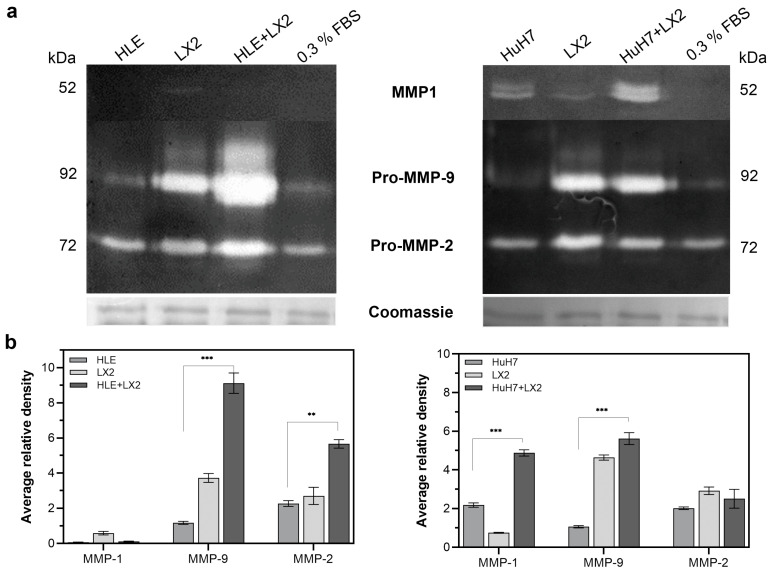
Abundance of MMP-2 and MMP-9 in the media of HLE, HuH7, and LX2 and the direct co-cultures HLE+LX2 and HuH7+LX2. MMP-9 was significantly induced in HLE+LX2 and MMP-1 was significantly induced in HuH7+LX2 compared to the respective monocultures. (**a**) Bands of zymography assay and (**b**) corresponding densitometric evaluation normalized to Coomassie staining. Data points represent mean ± SD, n = 3; ** *p* < 0.01; and *** *p* < 0.001.

Matrix metalloproteases secreted into the supernatants of single cultures and co-cultures were detected by zymography. Pro-MMP-2 was the dominant protease in HLE cells whereas LX2 expressed more pro-MMP-9. Pro-MMP-9 was markedly induced in the HLE+LX2 co-culture whereas the co-culture of HuH7 and LX2 resulted in the activation and upregulation of MMP-1 (Figure 6).

### 2.5. The Expression of Integrins and Their Cooperation with Adhesive Glycoproteins Are Hepatoma Cell Type-Dependent

**Figure 7:**
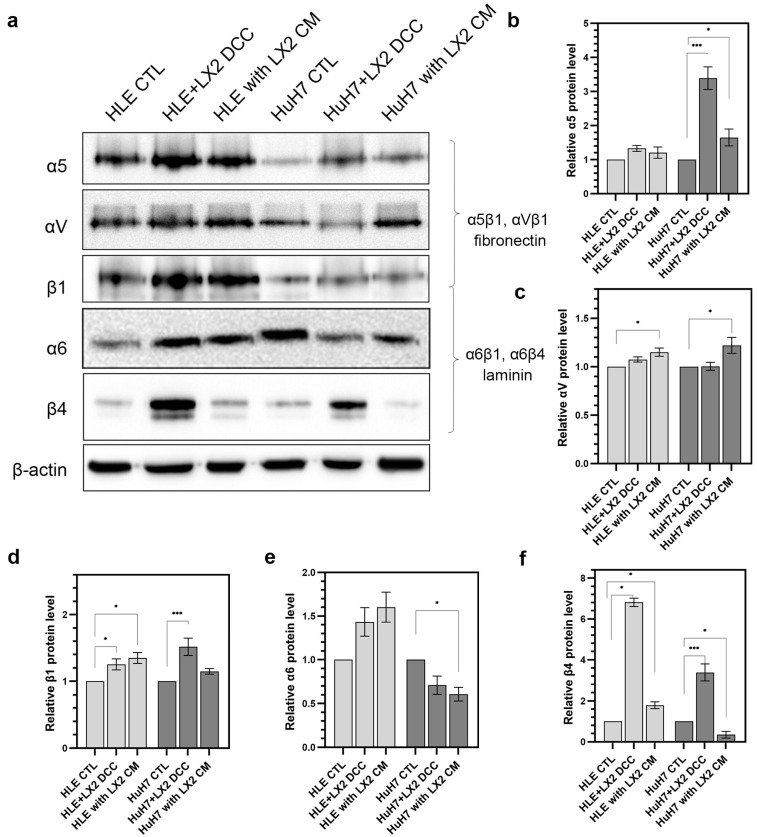
Expression of integrins that bind to laminin and fibronectin in HLE and HuH7 cells, alone or in co-culture with LX2 fibroblasts. (**a**) Immunoblots and (**b**–**f**) corresponding densitometry graphs β-actin were used as a reference for protein loading and relative activity levels were compared to respective controls. Data points represent mean ± SD, n = 3; * *p* < 0.05; and *** *p* < 0.001.

Integrins are pivotal to the cooperation between cells and matrix proteins. Integrins are heterodimeric transmembrane receptors on the surface of epithelial cells that selectively bind to their matrix ligands and these interactions modulate cell behavior. Integrin–matrix interactions can facilitate the proliferation, migration, and invasion of tumor cells. Here, invasive HLE cells were compared with less aggressive HuH7 cells with respect to the expression of integrin components that bind to two adhesive glycoproteins, the basement membrane resident laminin, and the ECM protein fibronectin. Tumor cells were grown either alone, in direct co-culture with LX2 cells, or in the presence of LX2-conditioned media. Compared to the HLE cells grown alone, fibronectin receptors α5β1 and αVβ1 were upregulated both in direct and indirect co-culture whereas the laminin receptor α6β4 increased considerably in the direct co-culture only. In HuH7 cells, α5β1 was increased in direct co-culture whereas components of the laminin receptor did not show coordinate changes in expression (Figure 7).

### 2.6. Conditioned Medium of LX2 Cells Upregulates CXCL12 in the Hepatoma Cell Lines

**Figure 8:**
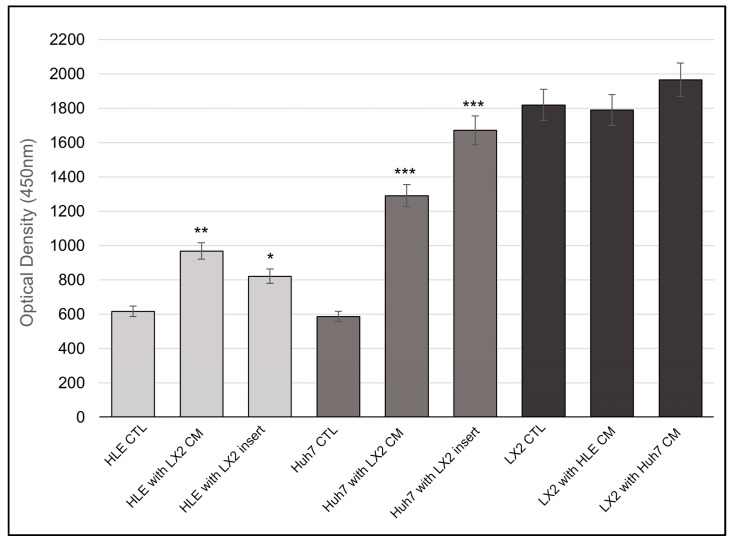
Effect of LX2 cell conditioned media on the expression of CXCL12 cytokine production of hepatoma cell lines. Data points represent mean ± SD, n = 3; * *p* < 0.05; ** *p* < 0.01; and *** *p* < 0.001.

HLE and HuH7 control cells were compared to those that were grown in a conditioned medium of LX2 as well as to those that shared medium with LX2 cells through co-culture inserts. Conversely, LX2 cells were grown alone or in the conditioned medium of hepatoma cells. Following treatment with a conditioned medium or indirect co-culture, the medium was replaced and allowed to be conditioned by the treated cells alone and then CXCL12 was quantified in this conditioned medium. Initially, low concentrations of CXCL12 were found to be increased by 60% and 30% in the medium of HLE cells and by 100% and 150% in the medium of HuH7 cells upon treatment with LX2-conditioned medium and upon co-culture with LX2, respectively. High baseline secretion of CXCL12 by LX2 cells remained unaltered following treatment with hepatoma-conditioned media (Figure 8).

### 2.7. Hepatoma Cell Lines and Fibroblasts Communicate by EVs

#### 2.7.1. Hepatoma EVs Activate ERK1/2 and Inhibit GSK3 Function in LX2 Cells

**Figure 9:**
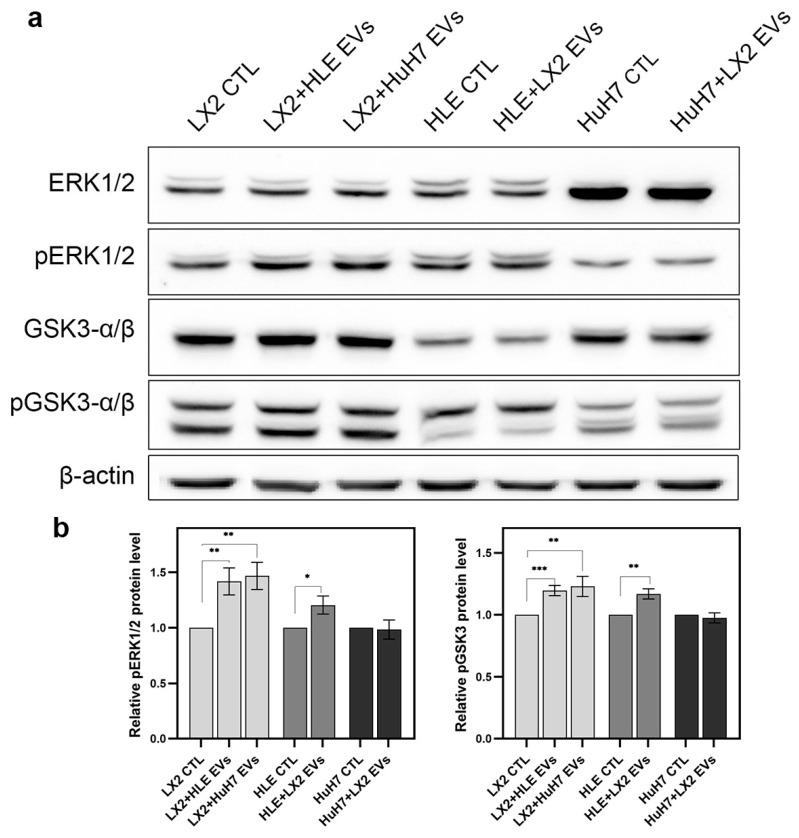
EVs from HLE and HuH7 upregulated pERK1/2 signaling and inhibited pGSK3-α/β in LX2 cells. A moderate pERK1/2 elevation was observed in HLE upon exposure to LX2 EVs. (**a**) Immunoblots and (**b**) corresponding densitometry graphs. β-actin housekeeping protein was used as a reference for protein loading and relative activity levels were compared to respective controls. Data points represent mean ± SD, n = 3; * *p* < 0.05; ** *p* < 0.01; and *** *p* < 0.001.

EVs isolated from the media of hepatoma cells upregulated pERK1/2 signaling and inhibited GSK3-α/β signaling in LX2 cells whereas LX2-derived EVs caused a moderate increase in pERK1/2 activity in HLE but no other effect was detected (Figure 9).

#### 2.7.2. LX2 EVs Alter the Cargo Composition of Hepatoma EVs

**Figure 10:**
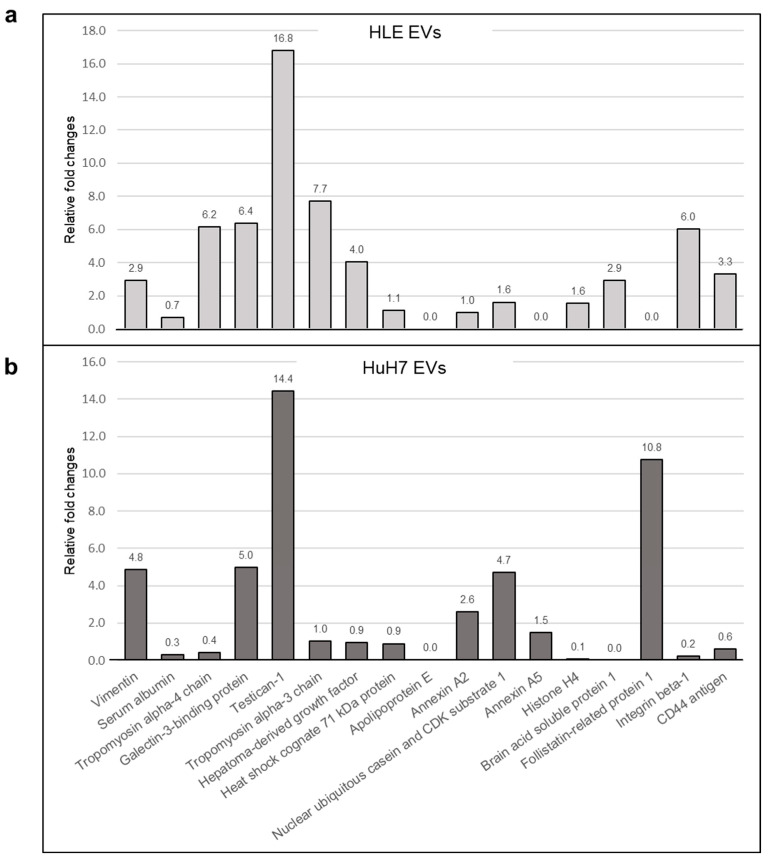
Exposure of (**a**) HLE and (**b**) HuH7 hepatoma cell lines to LX2-derived EVs changed the cargo composition of hepatoma EVs. The most prominent change was a more than tenfold increase in SPOCK1/testican-1. The relative fold change was calculated with reference to hepatomas with LX2 EVs relative to hepatoma CTL EVs.

First, control EVs were isolated from the conditioned media of untreated hepatomas and LX2 cells. Subsequently, hepatoma cell lines were exposed to LX2-derived EVs and LX2 was exposed to hepatoma-derived EVs for two days. Finally, the medium was replaced and the cells were allowed to produce their own EVs again. Both control and challenged EVs were analyzed by mass spectrometry. Treatment with LX2 EVs elicited marked changes in the cargo composition of hepatoma EVs (Figure 10). The most prominent change was a more than tenfold upregulation of SPOCK1/testican-1 in the EVs of both hepatoma cell lines. (A complete list is available in Supplementary Table S1). Furthermore, conversely, the treatment with hepatoma EVs also resulted in notable modifications to the cargo composition of LX2 EVs (detailed results in Supplementary Figure S1).

**Figure 11:**
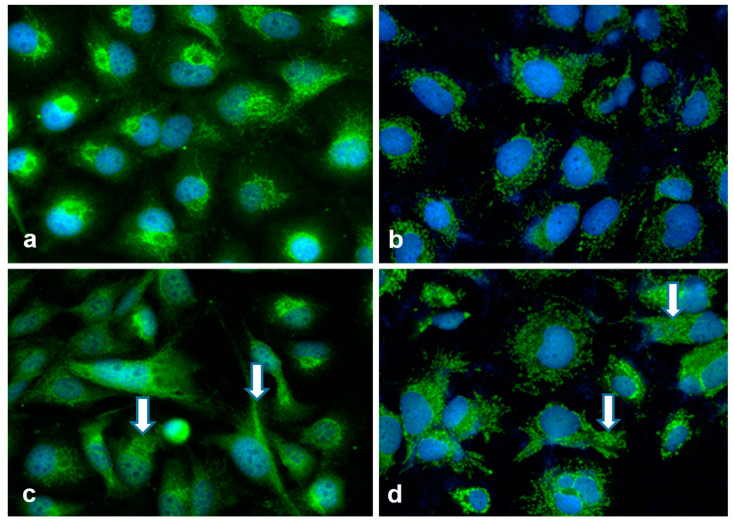
Morphology and SPOCK1/testican-1 immunostaining of hepatoma cells before (**a**,**b**) and after (**c**,**d**) treatment with LX2-derived EVs. (**a**,**c**): HLE and (**b**,**d**): HuH7. Green: testican-1 and blue: DAPI. Control hepatoma cells ((**a**), HLE and (**b**), HuH7) exhibited cytoplasmic testican-1 positivity. Upon exposure to LX2 EVs, these hepatoma cells underwent phenotypic changes and modifications in EV cargo. Their cytoplasm increased and elongation occurred while a small subset of HLE cells displayed intranuclear testican-1 positivity (arrows). Original magnification: 40×.

LX2 EVs also affected cell morphology and the distribution of testican-1 in hepatoma cells (Figure 11). Following exposure to LX2-derived EVs, previously round-shaped hepatoma cells grew elongated projections and testican-1 that displayed dominantly perinuclear unilateral localization in control HLE cells became more evenly dispersed in the cytoplasm. Testican-1 also appeared in the nucleus of HLE cells after LX2 EV treatment.

#### 2.7.3. LX2 EVs Modify the Expression of miRNA of Hepatoma Cell Lines

**Figure 12:**
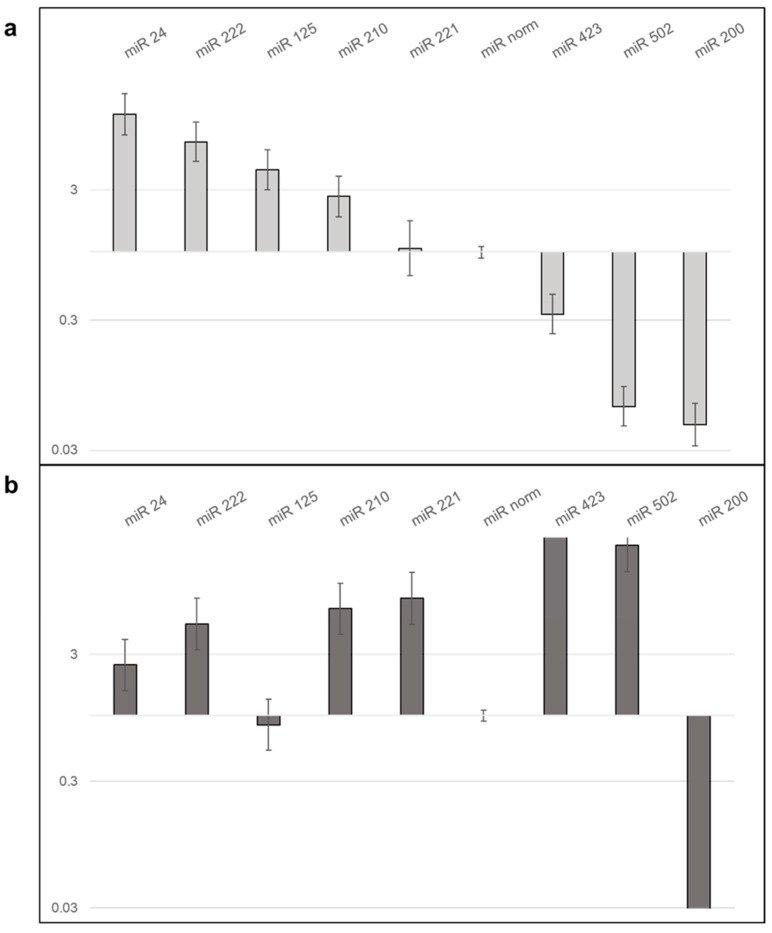
qRT-PCR validation of miRNAs shown to be differentially regulated by the TaqMan card in HLE (**a**) and HuH7 (**b**) upon treatment with LX2-derived EVs.

To further clarify the effect of LX2-derived EVs on hepatoma cells, miRNA expression was profiled in control and LX2 EV-treated hepatoma cells using a TaqMan array. Out of 373 miRNAs on the TaqMan card, 18 showed upregulation and 11 showed downregulation in HLE cells (comprehensive results from the TaqMan Array Card real-time PCR experiment are presented in Supplementary Table S2). Selected hits were validated by individual qRT-PCR assays (Figure 12 and Supplementary Table S3). Validation confirmed more than 3-fold upregulation of miR-24, 222, and 125 and a nearly 3-fold upregulation of miR-210 but failed to confirm upregulation of miR-221. Three downregulated miRs (423, 502, and 200) were confirmed by real-time PCR.

In HuH7, of 14 differentially regulated miR hits from the TaqMan card, miR-423 and 502 were validated by qRT-PCR and only downregulation of miR-200 was confirmed (detailed findings from the TaqMan Array Card real-time PCR experiment can be found in Supplementary Table S4). Albeit not detected by the TaqMan card, upregulation of miR-24, 210, 221, and 222, all known to be involved in the regulation of liver cancers, was demonstrated by individual qRT-PCR assays. Additional experimental data regarding miRNAs are provided in Supplementary Table S5 and Figure S2.

### 2.8. Conditioned Media of Tumor Cells and Fibroblasts Mutually Alter the Intermediary Metabolism

**Figure 13:**
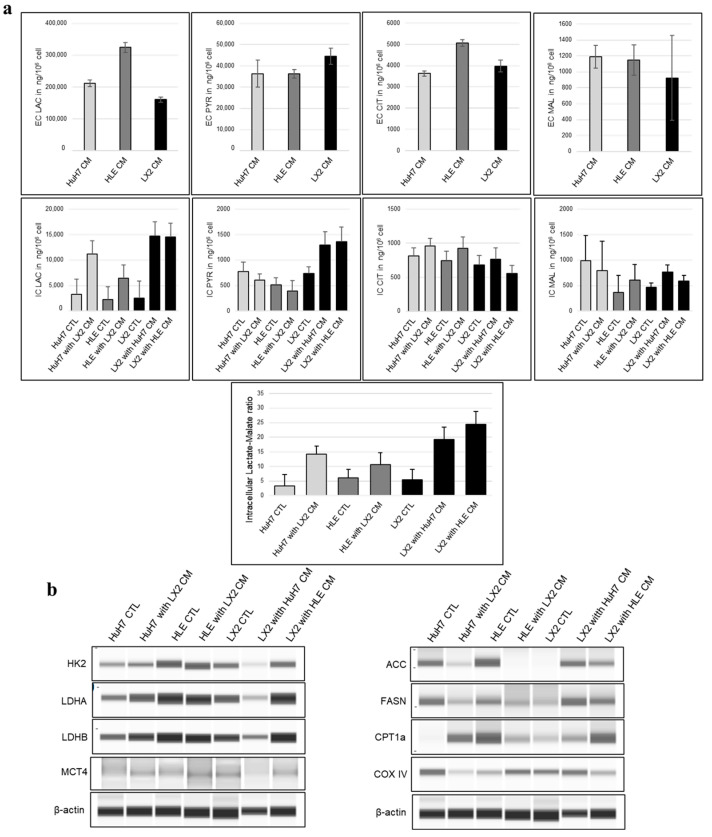
Metabolic changes in hepatoma and fibroblast cells after treatment with conditioned media. (**a**) Extracellular (after 48 h) and intracellular metabolite (lactate, pyruvate, citrate, and malate) concentration in control and treated hepatoma and fibroblast cell lines. (**b**) Metabolic activity related protein expression differences between control and treated cells. WES simple Western blot analyses were performed on selected proteins, with protein expression values normalized to β-actin. Raw WES simple electropherograms and corresponding densitometry graphs are available in Supplementary Materials, Figure S3. IC: intracellular, EC: extracellular LAC: lactate, PYR: pyruvate, CIT: citrate, MAL: malate, CTL: control, CM: conditioned medium, HK2: hexokinase 2, LDHA: lactate dehydrogenase A, LDHB: lactate dehydrogenase B, MCT4: monocarboxylate transporter 4, ACC: acetyl-CoA carboxylase, FASN: fatty acid synthase, CPT1A: carnitine palmitoyl transferase 1A, and COXIV: cytochrome c oxidase subunit IV.

Based on our metabolite concentration studies, the extra- and intracellular levels of certain metabolites and the amount of different metabolic proteins showed alteration in replaced conditioned media. The most aggressive HLE cell line released the highest level of lactate and citrate, while only minor differences were detected among the studied cells in the levels of pyruvate and malate release into the extracellular space (Figure 13a).

Lactate and pyruvate levels were increased intracellularly in LX2 cells after exposure to hepatoma-derived conditioned media, suggesting that medium-derived lactate is converted back to pyruvate by a reverse Warburg effect. LX2-conditioned media containing lower levels of lactate could increase intracellular lactate in the hepatoma cultures but no increase in pyruvate occurred, suggesting lactate efflux in hepatomas. Additionally to the increased citrate in the two hepatomas and the reduced lipid synthetic activity regarding decreasing fatty acid synthase (FASN) in parallel, acetyl-CoA carboxylase (ACC) protein expression may indicate a temporary decrease in fatty acid formation, while the increase in malate in HLE and LX2 cells may indicate increased OXPHOS activity in these cells. The lactate/malate ratio is a sensitive indicator of the metabolic state of cells which correlates with glycolytic activity. In all three cell lines, the intracellular (IC) lactate/malate ratio increased as a result of the conditioned media, which may indicate increased glycolysis. However, in LX2, this increase could be the reason for the uptake of lactate from the tumor cell-conditioned media (Figure 13a,b, and detailed LCMS data available in Supplementary Table S6).

We further investigated the metabolic changes induced by the conditioned media at the protein level using the WES simple technique, which revealed that lactate dehydrogenase B (LDHB), responsible for the lactate–pyruvate conversion, significantly increased in LX2 cells under the influence of the HLE medium. In addition, the protein levels of ACC and FASN, which are important in fatty acid formation, indicate a decrease in fatty acid formation in HCC cell lines, as we previously mentioned (raw WES simple electropherograms and corresponding densitometry graphs are available in Supplementary Materials, Figure S3) (Figure 13a,b). In contrast, these two proteins were elevated in LX2, suggesting fatty acid synthesis in the fibroblasts. We observed a parallel trend between the amount of cytochrome c oxidase subunit IV (COX IV) and IC-malate, which indicates a change in mitochondrial activity in HLE and LX2 cells after treatment with conditioned media (Figure 13a,b) and highlights potential symbiotic rewiring in hepatoma and fibroblasts induced by each other’s media.

## 3. Discussion

In spite of significant efforts in oncology, the slow decline in malignant disease incidence and mortality persists, particularly in challenging tumor types like pancreatic, lung, hepatocellular cancer, and melanoma [17]. A crucial lesson learned is that targeting cancer cells alone is insufficient, as non-tumorous cells within cancer tissue can also support tumor growth [17]. Harmless constituents like structural proteins, proteases, cytokines, and inflammatory cells can contribute to tumor development, confirming their role across all tumor types [3,5,18].

A key contributor to tumor progression Is the cancer-associated fibroblast (CAF), an integral part of the tumor stroma derived from normal fibroblasts that closely collaborates with tumor cells [19]. Various stromal cell types can transdifferentiate into CAFs during tumor development through mechanisms such as the influence of cancer-secreted extracellular vesicles (eVs) [20]. The outcome of CAF-tumor cell cooperation depends on tumor tissue origin and phenotype. Non-neoplastic cells can support or promote cancer progression, though CAFs have occasionally attenuated malignant behavior [21,22,23].

Our model explored the interaction between LX2 and tumor cells unveiling how fibroblasts contribute to tumor progression. LX2 modulated cancer cell metabolism and signaling and we anticipate that these are only a few of the many ways fibroblasts can facilitate tumor progression.

In summary, while our study provides valuable insights into multiple levels of interactions between fibroblasts and tumor cells, acknowledging its limitations is crucial to interpreting its findings and translating them into meaningful clinical implications. Further research in more complex models and in vivo settings is necessary to fully appreciate the implications of these findings in the context of cancer progression and treatment.

### 3.1. HLE and HuH7 as Co-Culture Partners Modeling Poorly Differentiated vs. Differentiated Hepatoma

Although the HLE cell line is markedly distinguished from HuH7 through its high proliferation rate, loss of cytokeratin, Transwell invasion capability, rapid migration in the wound healing assay, and the secretion of stromal adhesive glycoproteins, the responses of both cell lines to the conditioned medium or EVs from LX2 showed a surprising degree of overlap. Shared elements of the response included activation of pERK1/2 signaling, increased expression of NF-κB, and inhibitory phosphorylation of pGSK3-α/β as well as activation of β-catenin specifically in the case of HuH7 cells. Akt signaling, however, was differentially regulated in HLE vs. HuH7 as the conditioned medium of LX2 activated pAkt(T308) in HLE but pAkt(S473) in HuH7. These events may critically influence tumor progression.

The differentiation status of hepatoma cell lines also affected the extracellular matrix components they secreted into the medium in response to stimulation by LX2. The media of co-cultured LX2 and HLE cells contained increased amounts of laminin, type IV collagen, CD44, and shed syndecan-1. In contrast, co-cultured LX2 and HuH7 cells secreted fibronectin, thrombospondin, and type IV collagen and tended to retain syndecan-1 on the plasma membrane. Consistently, HLE cells preferentially expressed laminin-binding integrins, whereas HuH7 cells predominantly expressed fibronectin-binding integrins on their cell surface. Of note, similar differentiation-related tendencies in stromal laminin and fibronectin production were observed in cervical cancer [24].

### 3.2. Conditioned Medium of LX2 Cells Upregulated CXCL12 Expression in Hepatoma Cell Lines

Whereas conditioned media from hepatoma cells did not facilitate the CXCL12 expression in LX2 cells, LX2 significantly stimulated CXCL12 production of hepatomas, especially of HuH7, both in direct contact and via conditioned medium. CXCL12 concentration in the medium of untreated LX2 was twice as high compared to untreated hepatomas, indicating that fibroblasts were the major producers of this cytokine in this system. However, it should be noted that not only hepatoma cells but also hepatocytes have the capability to secrete the cytokines. Upon binding to its CXCR4 receptor, the cytokine participates in several cancer-related signaling pathways, such as PI3K, Akt, NFκB, and MAPK, all of which were also detected in our experiments [25,26,27]. Our study shows that the cooperation between LX2 cells and hepatomas increases the total level of CXCL12, potentially promoting the activity and aggressiveness of cancer cell lines through the CXCL12 ligand–CXCR4 receptor interaction [28]. However, it is important to note that further investigation is needed to confirm this hypothesis.

### 3.3. EVs as New Messengers between Hepatomas and Fibroblast Communication

The secretion of EVs is a regular activity in both normal and compromised cells. EVs are either released by exocytosis of multivesicular bodies or by plasma membrane shedding [29]. Their size and content may vary depending on the originating cell and they can be harnessed for diverse applications such as drug delivery, biomarker identification, early detection of tumors, cancer diagnosis, and disease management [29,30,31].

EVs isolated from the conditioned media of both hepatoma cell lines stimulated the pERK1/2 in LX2 cells and promoted the inactivation of GSK3-α/β. EVs from LX2 cells, on the other hand, modestly activated pERK1/2 in HLE cells but no other effect was detected.

Some of our most noteworthy results were obtained when hepatoma cell lines were exposed to LX2-derived EVs and then allowed to produce their own treatment-modulated EVs. The abundance of more than 100 proteins was analyzed by quantitative mass spectrometry to assess the effect of LX2 EV treatment on the protein composition of hepatoma-derived EVs. Among the proteins with increased EV abundance, we identified SPOCK1/testican-1, a proteoglycan increasingly accepted as oncogenic [32,33,34] that displayed over 10-fold elevation in the treatment-modulated EVs of both hepatoma cell lines. High expression of testican-1 was demonstrated in hepatocellular carcinomas, particularly in those associated with hepatitis C virus (HCV) [35], and in serous ovarian cancer [36], where testican-1 was also detectable in the sera of cancer patients. Future research should clarify the role of testican-laden circulating EVs in cancer patients.

### 3.4. EVs Isolated from the Hepatoma Cell Lines Contain Regulatory miRNAs

In the past two decades, EVs and miRNA in liquid biopsies have been intensively explored for their potential in the early detection of liver and other cancers [37,38,39]. Here, we addressed the question as to whether EVs isolated from the conditioned media of hepatoma cell lines contain miRNAs responsible for liver cancer progression. Due to technical challenges, only a few studies have so far analyzed the correlation of EVs and their miRNA cargo.

In this work, a large-scale analysis of miRNAs was carried out using the Megaplex primer pools and the TaqMan Array Card technology to find miRNAs already known to be implicated in the regulation of hepatoma biology. Of 370 potential targets, our final analysis confirmed 4 upregulated (miR-24, -222, -125, and -210) and 3 downregulated (miR-423, -502, and -200) miRNAs in the EVs of HLE as well as 2 upregulated (miR-423 and -502) miRNAs and 1 downregulated (miR-200) miRNA in HuH7-derived EVs. Intriguingly, some of these miRNAs were differentially regulated in the two hepatoma cell lines. Although we were unable to detect miR-24, 222, 125, and 201 in the large-scale screen of HuH7 EVs, the same miRNA species could be amplified by single RT-qPCR assays. miR-24 is known to actively contribute to liver cancer progression by inhibiting p53 and stimulating Sox7 [40,41]. miR-125-5p was detected earlier in the circulating EVs of HCV-induced liver cancer and miR125b-5p inhibiting TXNRD1 acting as a tumor suppressor in hepatocellular carcinoma [42,43]. miRNA-221 and -222, involved in the regulation of the Akt-mTOR signal transduction pathway [44,45], were both identified in our samples and had been found previously in circulating EVs of liver cancer patients where they were associated with poor prognosis.

The set of miRNAs detected in our EV samples largely overlapped with those reported in a paper discussing miRNAs dysregulated in the liver cancer [46]. The notion that these miRNAs regulate the Wnt/β catenin, MAPK, and Akt pathways is in good accordance with our results. miR-423, another miRNA known from human HCC, was downregulated in HLE EVs but upregulated in HuH7 EVs according to our large-scale array [47]. The same was observed for miR-502, which had been identified in multiple bioinformatics screens as a miR characteristic of hepatocellular cancer. miR-200, albeit another well-known regulator of liver cancer whose lack of expression can indicate a better prognosis, does not seem to be a resident miRNA of activated hepatoma-derived EVs [48,49].

### 3.5. Modulation of Metabolism by HCC–Fibroblast Interactions

Cancer-associated fibroblasts as major cellular constituents of the tumor stroma play significant roles in tumor growth, invasion, and metastasis. Due to their heterogeneous properties and diverse origin, CAFs can either support or suppress tumor development [50,51,52]. In recent years, a growing body of data has accumulated on the relationship between tumors and stroma, including interactions at the metabolite level [53,54,55]. In our study, changes in metabolite concentrations and protein expression upon treatment with conditioned media were investigated in HCC and fibroblast cell lines using LC-MS and WES simple techniques to assess alterations in metabolic state and the direction of metabolic processes. Our extracellular medium analysis revealed that the aggressively growing HLE cell line exhibited the most elevated levels of lactate and citrate, thus emphasizing its distinct metabolic profile. In response to the conditioned medium of hepatoma cells, we observed an increase in intracellular lactate and pyruvate levels in LX2 cells. This suggests that lactate derived from the medium is converted back to pyruvate through a reverse Warburg effect. Additionally, our results showed that the LX2-conditioned medium induced an increase in intracellular lactate levels in the hepatoma cultures. Concordantly, Lu et al. found that CAFs promote glycolysis via EVs in HCC cells [56]. The hepatoma cells demonstrated an elevation in citrate levels, implying a transient reduction in fatty acid formation. Conversely, the increase in malate levels observed in HLE and LX2 cells suggests an upregulation of OXPHOS activity within these cells. The lactate/malate ratio showed an increase in all three cell lines after exposure to the conditioned medium, which may indicate increased glycolysis in hepatoma cells and increased lactate uptake in LX2 cells which correlate with others’ observations [57,58]. In connection with the previous, LDHB, responsible for lactate–pyruvate conversion, showed a notable increase in LX2 cells under the influence of HLE medium. Additionally, changes in ACC, FASN, and intracellular citrate levels indicated a decrease in fatty acid formation within both tumor cell lines when exposed to LX2-conditioned media. In contrast, LX2 cells displayed elevated levels of ACC and FASN, suggesting active fatty acid synthesis in the fibroblasts. CAFs could exhibit augmented lipolysis, which contributes to the provision of lipid droplets as a fuel source for cancer cells. The lipids produced by fibroblasts and released into the medium can provide the tumor cells with the necessary lipids for building membranes and promoting tumor growth. Importantly, this process does not require the tumor to expend energy since the lipids are readily available for uptake [59,60]. In addition, the miRNAs identified in our extracellular vesicle samples also play a role in the regulation of fatty acid metabolism in previously reported studies [61,62]. We also observed a parallel correlation between the quantity of COX IV and intracellular malate, indicating changes in OXPHOS in both HLE and LX2 cells following treatment with the conditioned media. Metabolic coupling can be influenced by signaling pathways such as HIF1-α, NF-κB, and TGF-β which promote glycolysis, oxidative stress, and other cellular processes [63,64,65,66,67]. Our study concluded that quantitative changes in metabolites can provide valuable information about the metabolic state of cells and changes in metabolic processes upon the addition of conditioned media. Furthermore, our results hint at correlations between changes in metabolites and the quantities of specific metabolic proteins, although further studies are needed to confirm these relationships. In summary, the current study elucidates novel details of HCC–fibroblast interplay including coordinate changes in the metabolic state.

### 3.6. Cancer–Stromal Interactions in Hepatocellular Carcinoma: Insights, Limitations, and Future Prospects

While our study acknowledges certain limitations, it is important to recognize the scope of our investigation. We focused primarily on hepatoma cells HLE and HuH7, which, while informative, may not fully capture the diverse spectrum of hepatocellular carcinoma. Additionally, it is worth noting that the LX2 cell line, although commonly referred to as cancer-associated fibroblasts (CAFs), is an immortalized fibroblast line. However, it serves as a valuable model for initial investigations due to its well-characterized properties and ease of use. Future studies should consider the inclusion of a broader range of cell lines or patient-derived samples to enhance the study’s relevance.

The implications of our findings are substantial. They underscore the importance of tumor–stromal interactions in hepatocellular carcinoma, potentially leading to the identification of novel therapeutic targets and diagnostic biomarkers. The presence of liver cancer-specific miRNAs in hepatoma EVs offers promise for early diagnosis, deserving further exploration in clinical settings.

In the future, research should focus on assessing the functional consequences of the identified communication channels between hepatoma cells and fibroblasts. This involves investigating their impact on critical aspects of tumor biology, including growth, invasion, and therapy resistance. Additionally, efforts should be directed towards the clinical translation of our findings, exploring their applicability in the development of targeted therapies and diagnostic tools for hepatocellular carcinoma. To gain a more comprehensive understanding, researchers should consider the influence of additional stromal cell types and the dynamic nature of the tumor microenvironment in future investigations.

By addressing these limitations and pursuing these research directions, we aim to contribute to a deeper understanding of hepatocellular carcinoma biology and potentially improve clinical outcomes for patients.

## 4. Materials and Methods

### 4.1. Cell Cultures

The LX2 human hepatic stellate cell line was kindly provided by Dr. Scott Friedman. HuH7 and HLE were acquired from the Japanese Collection of Research Bioresources Cell Bank (Osaka, Japan). Cells were maintained in Dulbecco’s Modified Eagle’s Medium (DMEM) (Sigma Aldrich, St. Louis, MO, USA) with 1000 mg/mL glucose, 100 U/mL penicillin, 100 μg/mL streptomycin (Sigma Aldrich), and 10% (*v*/*v*) fetal bovine serum (Sigma Aldrich) in a 37 °C incubator with 85% relative humidity and 5% CO_2_ atmosphere.

### 4.2. Co-Culture Systems

The interaction between cells was studied by establishing various co-culture setups. Direct co-cultivation allowed physical contact between cells, whereas in indirect co-cultures cells were separated by a Transwell insert with a 0.8 μm pore size (#CLS3428, Sigma Aldrich), allowing only molecular communication.

Cells were seeded in 6-well plates (5 × 10^5^ cells/well density) alone or in direct co-cultures. A 1:1 mixture of cells was seeded and grown together in DMEM supplemented with 10% fetal bovine serum (FBS). Them, 72 h after seeding, the FBS content was reduced to 0.3% and cells were incubated for 24 h. Conditioned media (CM) and cell layers were then harvested and stored at −80 °C until use. Samples from direct co-cultures are indicated as hepatoma cell lines (HLE or HuH7) + LX2 stellate cells.

Indirect co-cultures were set up as follows: LX2 stellate cells were seeded in 6-well plates at a density of 2.5 × 10^4^ cells/well in DMEM with 10% (*v*/*v*) FBS. Hepatoma cells were placed in Transwell polyester membrane inserts (Sigma Aldrich) with 0.8 μm filter pore size at a density of 5 × 10^4^ cells/insert in DMEM with 10% (*v*/*v*) FBS. Then, 48 h after seeding, hepatoma cells containing inserts were placed in fibroblast-containing wells followed by refresh media supplemented with 10% (*v*/*v*) FBS. Subsequently, 72 h later, the FBS content was reduced to 0.3% (*v*/*v*) for another 24-h incubation period. CM and cells were then collected and frozen for later use.

### 4.3. Cell Proliferation Assay

The sulforhodamine B (SRB) assay (Sigma Aldrich) was used for the determination of HLE, HuH7, and LX2 cell density. Cells were seeded into 96-well plates at a density of 3000 cells/well. After 24 to 96 h of growth, cells were fixed with 10% (*w*/*v*) trichloroacetic acid for 60 min, washed in cold tap water, and dried for 24 h at 4 °C. Cells were then stained with 0.4% (*w*/*v*) SRB dissolved in 1% (*v*/*v*) acetic acid for 20 min and washed repeatedly with 1% (*v*/*v*) acetic acid to remove excess dye. The protein-bound dye was dissolved in 150 μL 10 mM TRIS (unbuffered). Optical density was measured at 570 nm using a Labsystems Multiscan MS 352 plate reader (Labsystems, Vantaa, Finland). The doubling time of cells was calculated from the log phase of their growth curves measured by the SRB assay at 24, 48, 72, and 96 h after seeding.

### 4.4. Chemotaxis Assay

To investigate the migration of HLE, HuH7, and LX2 cells, a 48-well micro chemotaxis chamber (Neuro Probe, Gaithersburg, MD, USA) was used with an 8 μm pore size polycarbonate membrane filter (Whatman, GE Healthcare Bio-Sciences Corp., Piscataway, NJ, USA). Fifty μL of cell suspension (5 × 10^4^ cells/mL in medium containing 10% FBS) was dispensed into the upper chamber. Matrigel (Sigma Aldrich) diluted in serum-free medium at 25 μg/mL was dispensed into the bottom chamber as the chemoattractant to simulate the extracellular matrix. After 4 h of incubation at 37 °C in a humidified atmosphere with 5% CO_2_, the chamber was disassembled and the cells on the upper side of the membrane were dislodged. The membrane was fixed in methanol and either stained with toluidine blue (Sigma Aldrich) or immunofluorescent staining for α-SMA was performed.

### 4.5. Wound Healing Assay

HLE, HuH7, and LX2 cells were plated on coverslips in a 6-well plate and incubated at 37 °C with 85% relative humidity and 5% CO_2_. At 95% culture confluency, cells were incubated with a complete medium containing 10 μg/mL mitomycin C (Merck KGaA, Darmstadt, Germany) for 3 h, followed by two washes with phosphate buffered saline (PBS). Sterile 200 μL pipette tips were utilized to create a scratch in each well. Cells were washed with PBS three times and then cultured in a fresh complete growth medium containing 1% (*v*/*v*) FBS. At 0-, 3-, 6-, and 24-h time points, slides were fixed using methanol, stained with H and E, and scanned using a Pannoramic P1000 scanner (3DHISTECH Ltd., Budapest, Hungary).

### 4.6. Immunofluorescence

Immunofluorescence staining was performed for the detection of vimentin, α-SMA, cytokeratin, and testican-1. Sterile 24 × 24 mm coverslips were placed into 6-well plates and cells were seeded at a density of 2.5 × 105/well and grown to 80% confluency while receiving conditioned media or EV treatment. The coverslips were washed by submerging them into ice-cold 1× Tris-buffered saline (TBS) and fixed in ice-cold methanol and acetone for 10 min and 1 min, respectively. The dried coverslips were stored at −20 °C or used immediately. Coverslips were washed three times in 1× PBS and then blocked with 5% (*w*/*v*) BSA (Sigma Aldrich) in 1× PBS for 1 h at room temperature. Incubation with the primary antibodies, diluted in 1% BSA, was performed overnight at 4 °C in a wet chamber. The following day, after washing three times with 1× PBS, the coverslips were incubated in the dark with the appropriate fluorescent secondary antibodies, diluted 1:200 in 1% (*w*/*v*) BSA with 10% (*v*/*v*) human serum, and were counterstained with 4′-6′-diamidino-phenylindole (DAPI, Sigma) for one hour. After three washes with 1x PBS, coverslips were mounted on non-autofluorescent slides using an aqueous fluorescence mounting medium (Sigma Aldrich). Pictures were taken using a Nikon Eclipse E600 fluorescent microscope and Lucia Cytogenetics version 1.5.6 software or 3DHISTECH Pannoramic Confocal scanner (3DHISTECH Ltd., Budapest, Hungary). Details of antibodies and their appropriate dilutions are found in Supplementary Table S7.

### 4.7. Expression Analysis of Proteins by Western Blot, Dot Blot, and WES Simple Capillary Immunoassay

To conduct protein analysis using Western blot and WES simple methods, protein extracts from 10^6^ lysed cells were quantified using the Bradford assay (BioRad). For the Western blot, proteins were separated using SDS-PAGE and transferred to a PVDF membrane using a wet technique (BioRad, Hercules, CA, USA). The membrane was then incubated with specific antibodies. Ponceau staining and anti-β-actin (Sigma Aldrich) were used as a loading control. Biotinylated secondary antibodies were incubated with an avidin–HRP complex (Vectastain Elite ABC Kit, Vector, Milwaukee, WI, USA) and the signal was visualized using enhanced chemiluminescence (ECL Western blotting substrate using C-digit appliance Thermo Fisher Scientific Inc., Waltham, MA USA). The band density of each protein of interest was normalized to its corresponding β-actin band.

Protein expression was also analyzed using a WES system (ProteinSimple, Biotechne, 004-600, Minneapolis, MN, USA) following the manufacturer’s instructions. Proteins ranging from 12–230 kDa were separated using the ProteinSimple #SM-W004 separation module. Depending on the species of the primary antibodies, the anti-rabbit detection kit (#DM-001, ProteinSimple) or the anti-mouse detection kit (#DM-002, ProteinSimple) was applied. Samples were diluted to an appropriate concentration (0.2 or 1 μg/μL depending on the applied primary antibody) in 0.1× sample buffer (diluted from ‘10× sample buffer’ from the separation module) and boiled at 95 °C for 5 min. The samples were then diluted with fluorescent master mix 1:4 and added to the plate along with the antibody diluent (ProteinSimple, Biotechne, #042-203), primary and secondary antibodies, and the chemiluminescent substrate. The device settings used for analysis included stacking and separation at 395 V for 30 min, blocking reagent for 5 min, primary and secondary antibodies both for 30 min, and luminol/peroxide chemiluminescence detection for 15 min (exposure times between 1 and 512 s). Electropherograms were analyzed and automatic peak detection was revised manually if necessary.

For dot blot analyses, 200 μL cell supernatant per well was vacuum-filtered on a PVDF membrane using a 96-well Minifold-Vakuum-Filtrations System SRC-96 (Schleicher and Schuell, Dassel, Germany). Ponceau staining was performed to assess the total amount of protein per dot. Endogenous peroxidase was blocked by incubating the membrane in 1% (*v*/*v*) H2O2 for 10 min. Subsequently, the membrane was blocked with 5% (*w*/*v*) BSA in TBS and then incubated with primary antibodies diluted in 1% BSA (TBS) overnight at 4 °C. Appropriate HRP-conjugated secondary antibody was used for 1 h, dots were detected by a SuperSignal west pico chemiluminescent substrate kit (Thermo Fisher Scientific Inc.), visualized, and analyzed using the same method as for Western blot. A detailed list of the antibodies used in this study is presented in Supplementary Table S7.

### 4.8. Zymography Assay

The proteolytic activity of cell culture media was measured by zymography. In brief, 20 μL of serum-free conditioned media was loaded onto each well in 2× Laemmli loading buffer under non-reducing conditions (without β-mercaptoethanol and boiling) on 7.5% modified acrylamide gels containing 3 μg/mL casein (α-casein from bovine milk, Sigma Aldrich), 5 μL/mL fibronectin (Sigma Aldrich), and 10 μL/mL matrigel (#E1270, ECM Gel from Engelbreth-Holm-Swarm murine sarcoma, Sigma Aldrich). After electrophoresis at 200 V for 30 min, the gels were washed with 2.5% (*v*/*v*) Triton X-100 for 30 min and incubated for 18 h in a buffer solution containing TRIS (pH 7.5) and 10 mM CaCl_2_ at 37 °C. After fixing in methanol–acetic acid–water (5:1:4) for 30 min, the gels were stained in 0.1% (*w*/*v*) Coomassie Brilliant Blue R-250 solution (dissolved in the fixation buffer) for 30 min. Areas of enzyme activity appeared as transparent bands on the blue background. The intensity of the bands was determined by densitometry using the free ImageJ (Version 1.50b, NIH, Bethesda, MD, USA) software.

### 4.9. ELISA

CXCL12/SDF-1α levels in the supernatant of mono- and co-cultured cells were measured by the human CXCL12/SDF-1α ELISA kit (R and D Systems, Minneapolis, MN, USA) using 100 μL conditioned cell medium per sample. The assay was carried out according to the manufacturer’s instructions. Each measurement was performed in duplicate and the mean values were used for statistical analysis. ELISA plates were read at a wavelength of 570 nm using Labsystems Multiscan MS 352 plate reader (Labsystems, Finland).

### 4.10. Extracellular Vesicle (EV) Isolation, Total EV RNA Isolation, and miRNA Expression Profiling

Prior to isolation, cells were washed three times with PBS, and EV production was allowed to take place for 24 h in a serum-free DMEM medium to avoid contamination of the preparations with EVs and proteins present in fetal bovine serum.

EVs were isolated using total exosome isolation reagent (#4478359, Thermo Fisher Scientific Inc., Waltham, MA, USA) following the instructions of the manufacturer. In brief, 30 mL of clear media was mixed thoroughly with 15 mL reagent in sterile 50 mL tubes and incubated at 4 °C overnight. The next day, the mixture was ultracentrifuged in an Optima MAX-XP benchtop ultracentrifuge with MLA-55 rotor (Beckman Coulter Inc., Brea, CA, USA) at 100,000× *g* for 70 min at 4 °C to pellet EVs. The EV pellet was re-suspended in PBS and stored at −80 °C until use. The purity of the isolate was determined using positive (CD63) and negative (calnexin) markers with Western blot analysis (Supplementary Figure S4). Further characterization of EVs released by hepatoma cells (HLE, HuH7) and LX2 stellate cells can be found in Supplementary Figure S5.

RNA from EVs was isolated using a total exosome RNA and protein isolation kit (#4478545, Thermo Fisher Scientific Inc.) following the manufacturer’s protocol. The yield and purity of RNA were determined using a NanoDrop ND-1000 spectrophotometer (NanoDrop Technologies, Wilmington, DE, USA). Small RNA enrichment was not used.

Total RNA samples were reverse transcribed with a TaqMan™ MicroRNA reverse transcription kit (#4366596, Thermo Fisher Scientific Inc.). miRNA expression profiling was performed using the RT-qPCR-based TaqMan™ array human MicroRNA A cards v2.0 (#4398965, Thermo Fisher Scientific Inc. Waltham, MA USA) according to the manufacturer’s protocol. The 2^−ΔΔCT^ method was used to evaluate relative changes in miRNA expression.

### 4.11. Chromatography and Mass Spectrometry for Proteomics Analyses

Proteins were precipitated in 9× volume ice-cold ethanol at −20 °C overnight. Pellets were washed twice with ice-cold ethanol and dissolved in 8 M urea in 50 mM ammonium bicarbonate. Protein concentration was determined and a 20 µg protein/sample was digested. First, dithiothreitol (DTT) was added at a final concentration of 5 mM and incubated at 37 °C for 30 min. Alkylation was performed in the dark at room temperature for 30 min in the presence of 10 mM iodoacetamide (IAA). Samples were diluted 10-fold with 50 mM ammonium bicarbonate and 1 µL 250 ng/µL trypsin/Lys-C mix (Promega, Madison, WI, USA) was added and incubated at 37 °C for 1 h. Next, 1 µL 1000 ng/µL trypsin (Promega, Madison, WI, USA) was added and the samples were incubated for another 2 h. Digestion was quenched by the addition of 1 µL formic acid. Desalting was performed on Pierce C18 spin columns (Thermo Fisher Scientific, Waltham, MA, USA). Samples were dissolved in 20 µL solvent (98% water, 2% acetonitrile, and 0.1% formic acid) out of which 0.5 µL was subjected to nanoLC-MS/MS analysis using a Dionex Ultimate 3000 RSLC nanoLC (Dionex, Sunnyvale, CA, USA) coupled to a Bruker Maxis II Q-TOF (Bruker Daltonik GmbH, Bremen, Germany) via a CaptiveSpray nanoBooster ionization source. Peptide separation was achieved on an Acquity M-Class BEH130 C18 analytical column (1.7 µm, 75 µm × 250 mm Waters, Milford, MA, USA) using gradient elution (4–50% eluent B in 120 minutes) following trapping on an Acclaim PepMap100 C18 (5 µm, 100 µm × 20 mm, Thermo Fisher Scientific, Waltham, MA, USA) trap column. Solvent A consisted of water + 0.1% formic acid while Solvent B was acetonitrile + 0.1% formic acid. The spectra were obtained at a fixed cycle time of 2.5 s with MS spectra scanned at a rate of 3 Hz and collision-induced dissociation (CID) was performed at 16 Hz for abundant precursors and 4 Hz for low abundance ones. Internal calibration was conducted at the beginning of each measurement using sodium format clusters and data were automatically recalibrated using Compass Data Analysis 4.3 software (Bruker Daltonik GmbH, Bremen, Germany).

Experimental data were submitted to the MassIVE data repository with the ID: MSV000090112. Protein identification was conducted using ProteinScape 3.0 software from Bruker Daltonik GmbH. Only proteins identified with a minimum of two unique peptides and a 1% false discovery rate (FDR) were considered for acceptance. For label-free quantitation, MaxQuant software version 1.5.3.30 was used, with the database generated from the proteins previously identified with ProteinScape, using the default settings of the program [68].

### 4.12. Metabolite Analysis by Liquid Chromatography Mass Spectrometry

The method used for the extraction of intracellular and extracellular metabolites including malate (MAL), citrate (CIT), lactate (LAC), and pyruvate (PYR) was modified based on Szoboszlai et al. [69]. Briefly, cells were snap-frozen in liquid nitrogen and extracted using a MeOH-H_2_O-chloroform mixture (9:1:1) at 4 °C. The supernatants were stored at −80 °C after centrifugation (15,000× *g*, 4 °C, 10 min). The concentrations of metabolites were determined using calibration curves obtained by diluting analytical grade standards in the range of 0.5–50 µM. A PerkinElmer flexar FX10 ultra-performance liquid chromatograph (Waltham, MA, USA) coupled with a Sciex 5500 QTRAP mass spectrometer (Toronto, ON, Canada)) was utilized for LC-MS analysis. Chromatographic separation was carried out on a Phenomenex Luna Omega C18 column (100 × 2.1 mm, 1.6 µm) (GenLab Ltd., Widnes, UK) using a mobile phase composed of water and methanol containing 0.1% (*v*/*v*) formic acid. The mass spectrometer was operated in negative electrospray ionization mode with the following settings: source temperature of 300 °C, ionization voltage of −4000 V, entrance potential of −10 V, curtain gas at 35 psi, gas1 at 35 psi, gas2 at 35 psi, and CAD gas at medium. Multiple reaction monitoring (MRM) mode was used for quantitative analysis.

### 4.13. Statistical Analysis

Data were analyzed using GraphPad Prism 9.1.2 (GraphPad Software, La Jolla, CA, USA) and Microsoft Excel v.2016 (Microsoft Corp., Redmond, WA, USA). For experiments with three biological replicates in each group, non-parametric statistical tests were employed. The Mann–Whitney U-test was used for comparisons between two groups in the case of Western blot, dot blot, array, gelatin zymography, and mass spectrometry (MS) data. For enzyme-linked immunosorbent assay (ELISA) data, one-way Kruskal–Wallis analysis was used for comparisons among multiple groups. Significance levels were indicated as * *p* < 0.05; ** *p* < 0.01; and *** *p* < 0.001.

## 5. Conclusions

**Figure 14:**
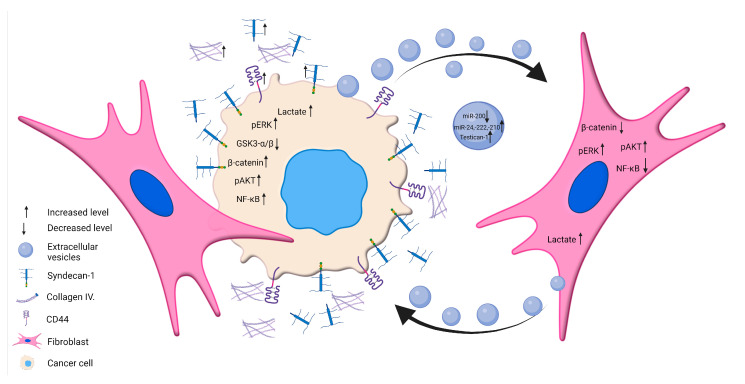
Overview of multiple levels of cancer cell–fibroblast communication. We investigated the communication between poorly and well-differentiated hepatoma cells and LX2 immortalized fibroblasts via soluble factors, metabolites, EVs, and miRNAs. LX2 fibroblasts, when co-cultured with HLE cells, exhibited increased production of laminin β1, type IV collagen, CD44, and shedding of syndecan-1, whereas the dominant matrix components in the HuH7/LX2 co-culture system included type IV collagen and cell surface syndecan-1. Hepatoma-derived EVs influenced the MAPK and Wnt signaling pathways in LX2 cells. LX2-derived EVs induced significant upregulation of testican-1 in the EV cargo of hepatoma cells. Hepatoma-derived EVs carried miRNAs that were differentially regulated upon interaction with LX2, could impact tumor progression, and may serve as diagnostic markers. Created with BioRender.com.

In this work, we characterized the multifaceted communication between hepatoma cells and LX2-immortalized fibroblasts. Authors are aware that LX2 is an immortalized, but not yet transformed, liver fibroblast. However, it already communicates with the tumor cells, facilitating their tumor-related functions. We co-cultured LX2 cells representing CAFs with aggressive HLE cells and better-differentiated HuH7 cells. HLE and HuH7 reacted differently to soluble and EV-borne signals from LX2 cells with regard to cell signaling pathways and the secretion of matrix components but both responded with upregulation of CXCL12. EVs from hepatoma cells carried HCC-specific regulatory miRNA species, which could be leveraged for diagnostics if confirmed in patient sera, and their EV cargo displayed over a 10-fold increase in SPOCK1/testican-1 abundance upon stimulation by LX2 EV. HCC cells and LX2 reciprocally altered each other’s metabolism in an interaction reminiscent of metabolic coupling. Thus, even in this simplistic in vitro model, we were able to shed some light on the versatility of intercellular cooperation between tumor and stromal cells (Figure 14).

## Data Availability

The proteomics measurement data have been submitted to the MassIVE repository under the submission number MSV000090112.

## Supplementary Materials

The supporting information can be downloaded at: https://www.mdpi.com/article/10.3390/ijms241813996/s1.
