# Supplementary material for: Dynamic Interplay in Tumor Ecosystems: Communication between Hepatoma Cells and Fibroblasts
Source: Int J Mol Sci. 2023 Sep 12;24(18):13996. doi: 10.3390/ijms241813996 (PMC10530979; doi:10.3390/ijms241813996)
Supplement: Supplementary file 1 [file ijms-24-13996-s001.zip › Supplementary_ijms-2558533.pdf]

Dynamic Interplay in Tumor Ecosystems: Communication between Hepatoma Cells and Fibroblasts

Gábor Petővári [1], Gábor Tóth [2], Lilla Turiák [2], Anna L. Kiss [3], Krisztina Pálóczi [4], Anna Sebestyén [1], Adrián Pesti [5], András Kiss [5], Kornélia Baghy [1], Katalin Dezső [1], Tibor Füle [6], Péter Tátrai [7], Ilona Kovalszky [1,*,†] and Andrea Reszegi [1,5,8,*,†]

[1] Department of Pathology and Experimental Cancer Research, Semmelweis University, Üllői út 26, H-1085 Budapest, Hungary
[2] MS Proteomics Research Group, Research Centre for Natural Sciences, Eötvös Loránd Research Network, Magyar Tudósok Körútja 2, H-1117 Budapest, Hungary
[3] Department of Human Morphology and Developmental Biology, Semmelweis University, Tűzoltó u. 58, H-1094 Budapest, Hungary
[4] Department of Genetics, Cell and Immunobiology, Semmelweis University, H-1085 Budapest, Hungary
[5] Department of Pathology, Forensic and Insurance Medicine, Semmelweis University, Üllői út 93, H-1091 Budapest, Hungary
[6] Thermo Fisher Scientific Inc., Váci út. 41-43, H-1134 Budapest, Hungary
[7] Charles River Laboratories Hungary, Irinyi József utca 4-20, H-1117 Budapest, Hungary
[8] Department of Pediatrics, College of Medicine, University of Florida, Gainesville, FL 32610, USA
* Correspondence: kovalszky.ilona@med.semmelweis-univ.hu (I.K.); andrea.reszegi@ufl.edu (A.R.); Tel.: +36-208250268 (I.K.)
† These authors contributed equally to this work.

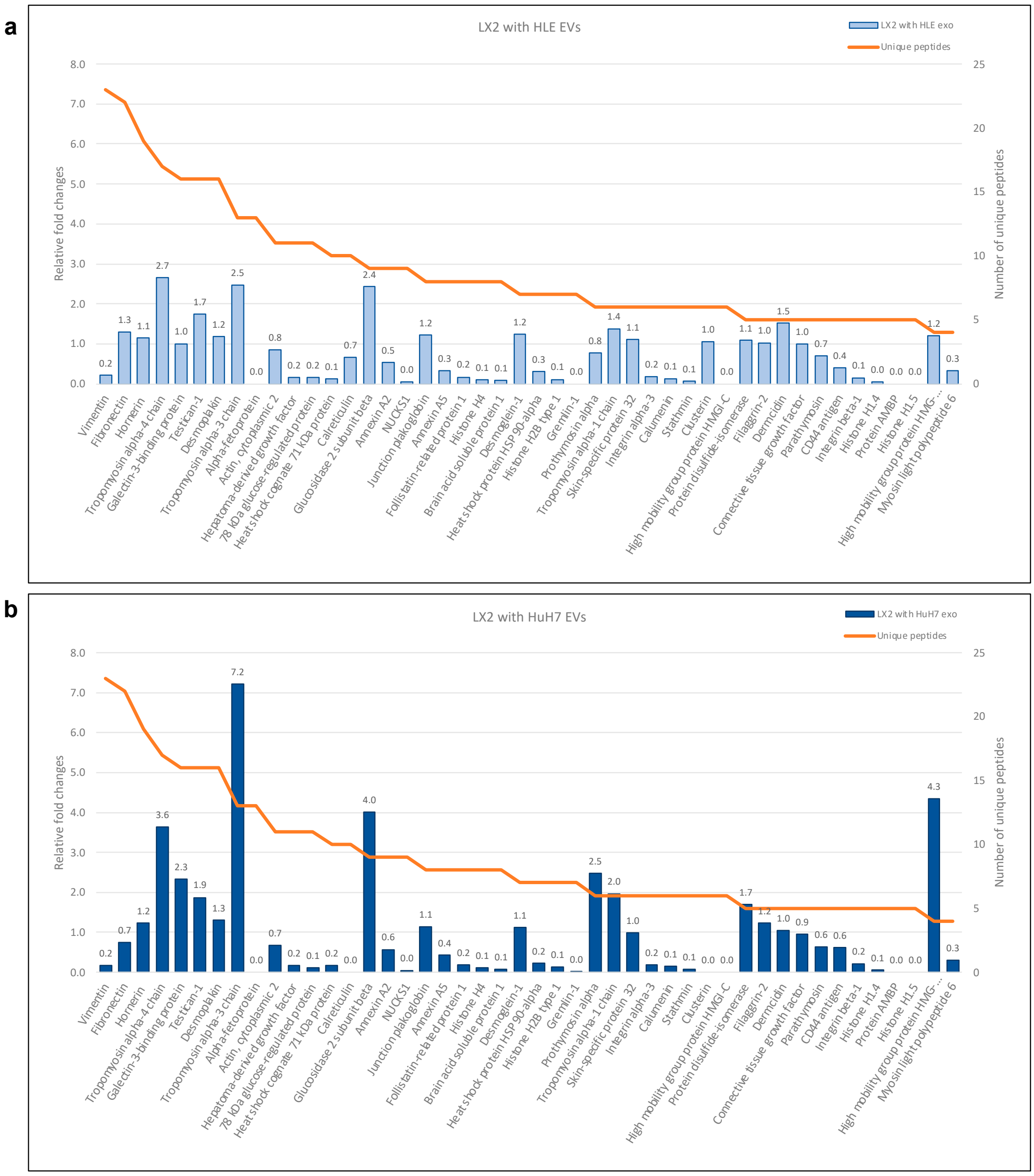

Figure S1. Exposure of LX2 fibroblast cell lines to hepatoma-derived extracellular vesicles (EVs) changed the cargo composition of LX2 EVs. Initially, control EVs were isolated from the media of untreated hepatomas and LX2 cells. Subsequently, LX2 fibroblast cell lines were exposed to hepatoma-derived EVs (a, HLE, b, HuH7) for a duration of 2 days. Following this, the media was replaced, allowing the cells to resume production of their own EVs. Both control and challenged EVs were subjected to mass spectrometry analysis. Notably, treatment with hepatoma EVs led to significant alterations in the cargo composition of LX2 EVs.

Table S1 MaxQuant protein quantitation results for describing the changes in different proteins (Excel sheet attached).

Table S2. Results table of TaqMan Array Card real-time PCR experiment -HLE Cells treated by EVs.

HLE x	Target Name	dd Cq	RQ
UP	**hsa-miR-222-3p-477982_mir***	**-4.04**	**16.39**
	**hsa-miR-24-3p-477992_mir***	**-3.49**	**11.20**
	**hsa-miR-125b-5p-477885_mir***	**-2.55**	**5.85**
	hsa-miR-423-3p-478327_mir	-2.51	5.71
	hsa-miR-31-5p-478015_mir	-2.51	5.68
	hsa-miR-181c-5p-477934_mir	-2.39	5.24

	hsa-miR-193a-5p-477954_mir	-2.12	4.35
	hsa-miR-21-5p-477975_mir	-2.01	4.04
	hsa-miR-181a-5p-477857_mir	-1.84	3.59
	hsa-miR-181b-5p-478583_mir	-1.68	3.21
	hsa-miR-186-5p-477940_mir	-1.47	2.78
	hsa-miR-17-5p-478447_mir	-1.31	2.48
	hsa-miR-500a-5p-478309_mir	-1.17	2.25
	hsa-miR-320a-478594_mir	-1.01	2.02
	**hsa-miR-221-3p-477981_mir***	**-0.91**	**1.87**
	hsa-miR-146a-5p-478399_mir	-0.80	1.74
	hsa-miR-342-3p-478043_mir	-0.67	1.59
	hsa-miR-361-5p-478056_mir	-0.58	1.50
DOWN	hsa-miR-501-5p-478142_mir	0.10	0.93
	**hsa-miR-423-5p-478090_mir***	**0.22**	**0.86**
	hsa-miR-2110-477971_mir	0.33	0.80
	hsa-miR-448-478105_mir	0.48	0.72
	hsa-miR-190a-5p-478358_mir	1.04	0.49
	hsa-miR-653-5p-479134_mir	1.04	0.49
	hsa-miR-380-3p-477854_mir	1.08	0.47
	**hsa-miR-502-3p-478348_mir***	**1.17**	**0.44**
	hsa-miR-374b-5p-478389_mir	1.29	0.41
	**hsa-miR-200a-3p-478490_mir***	**1.52**	**0.35**
	hsa-miR-153-3p-477922_mir	1.91	0.27

Total RNA samples extracted from cultured cells have been tested for expression profile of 377 micro-RNA (miRNA, miR) targets in TaqMan Array Card (TAC) real-time PCR. The miRNA profile of EV-treated HLE cells (HLE x) is compared to the control HLE cells' profile. Targets in the upper part of the table showing increased expression level (UP) compared to control are colored as orange; Targets in the lower part of the table showing decreased expression level (DOWN) compared to control are colored as green.

Only those miR targets listed in this table which show at least a 50% expression difference to control. The miR targets written in bold and labeled with (*) are selected for verification experiments. Target Name: human miRNA targets with assay IDs; ddCq: 'delta-delta-Cq' - normalized to control sample; RQ: Relative Quantity of the target miRNA compared to the control sample expression level used as reference; Analysis software: Thermo Fisher Connect, Relative Quantification module; Quantification Cycle (Cq) generation method: relative threshold cycle (CRT); Analysis criteria: amplification score (AmpScore) cutoff = 1; Cq confidence (CqConf) cutoff = 0,8; Cq cutoff = 33.

Table S3. Results Datasheet of real-time PCR verification experiment

Sample Name	Target Name	Cq Mean	Cq SD	dCq Mean	dCq SD	ddCq	RQ	RQ Min 2sd	RQ Max 2sd	RQ Min 1sd	RQ Max 1sd
HLE K	miR 125	29.9508	0.0435	1.2383	0.1776	0.0	1.0	0.7818	1.2791	0.8842	1.1310
HLE K	miR 502	28.1892	0.5522	-0.5233	0.5784	0.0	1.0	0.4485	2.2296	0.6697	1.4932
HLE K	miR 423	30.5584	0.0458	1.8459	0.1782	0.0	1.0	0.7811	1.2802	0.8838	1.1314
HLE K	miR 222	28.4180	0.1308	-0.2944	0.2162	0.0	1.0	0.7410	1.3495	0.8608	1.1617
HLE K	miR 221	22.4235	0.0384	-6.2890	0.1764	0.0	1.0	0.7831	1.2771	0.8849	1.1301
HLE K	miR 24	31.9272	0.0293	3.2147	0.1747	0.0	1.0	0.7850	1.2740	0.8860	1.1287
HLE K	miR 210	28.3317	0.0557	-0.3808	0.1810	0.0	1.0	0.7781	1.2851	0.8821	1.1336
HLE K	miR 200	29.9010	0.0977	1.1885	0.1980	0.0	1.0	0.7600	1.3158	0.8718	1.1471
HLE K	miR ref	28.7125	2.7548	0.0	0.0	0.0	1.0	1.0	1.0	1.0	1.0
HLE x	miR 125	24.7081	0.0293	-0.8616	0.2521	-2.1000	4.2870	3.0225	6.0805	3.5996	5.1056
HLE x	miR 423	26.6302	0.0135	1.0605	0.2508	1.5838	0.3336	0.2356	0.4723	0.2804	0.3969
HLE x	miR 502	31.3586	0.0250	5.7888	0.2517	3.9429	0.0650	0.0459	0.0922	0.0546	0.0774

HLE x	miR 222	22.4656	0.0282	-3.1042	0.2520	-2.8097	7.0115	4.9441	9.9433	5.8878	8.3497
HLE x	miR 221	19.1881	0.2467	-6.3816	0.3516	-0.0926	1.0663	0.6550	1.7360	0.8357	1.3606
HLE x	miR 24	25.2740	0.0768	-0.2958	0.2619	-3.5105	11.3963	7.9262	16.3856	9.5041	13.6651
HLE x	miR 210	23.7699	0.0741	-1.7999	0.2611	-1.4191	2.6742	1.8620	3.8408	2.2314	3.2048
HLE x	miR 200	31.1635	0.0999	5.5938	0.2696	4.4053	0.0472	0.0325	0.0686	0.0391	0.0569
HLE x	miR ref	25.5698	4.0066	0.0	0.0	0.0	1.0	1.0	1.0	1.0	1.0
HuH7 K	miR 125	35.7141	0.3732	2.4196	0.4277	0.0000	1.0	0.5527	1.8092	0.7435	1.3450
HuH7 K	miR 423	40.0000	0.0010	6.7055	0.2088	0.0000	1.0	0.7487	1.3356	0.8653	1.1557
HuH7 K	miR 222	36.7555	0.2448	3.4611	0.3217	0.0000	1.0	0.6402	1.5620	0.8001	1.2498
HuH7 K	miR 502	32.3180	0.1908	-0.9765	0.2828	0.0000	1.0	0.6757	1.4800	0.8220	1.2165
HuH7 K	miR 221	27.5271	0.0957	-5.7674	0.2297	0.0000	1.0	0.7273	1.3749	0.8528	1.1726
HuH7 K	miR 24	35.1619	0.2487	1.8674	0.3247	0.0000	1.0	0.6375	1.5686	0.7984	1.2524
HuH7 K	miR 210	35.6417	0.4128	2.3472	0.4626	0.0000	1.0	0.5266	1.8989	0.7257	1.3780
HuH7 K	miR 200	29.9432	0.0610	-3.3513	0.2175	0.0000	1.0	0.7397	1.3519	0.8601	1.1627
HuH7 K	miR ref	33.2945	3.3402	0.0	0.0	0.0	1.0	1.0	1.0	1.0	1.0
HuH7 x	miR 125	28.0972	0.0183	2.6717	0.3342	0.2521	0.8397	0.5283	1.3344	0.6661	1.0585
HuH7 x	miR 423	26.7888	0.0242	1.3633	0.3345	-5.3422	40.5661	25.5128	64.5014	32.1707	51.1524
HuH7 x	miR 222	26.4926	0.0653	1.0671	0.3400	-2.3940	5.2561	3.2807	8.4207	4.1526	6.6528
HuH7 x	miR 221	21.3885	0.0819	-4.0370	0.3436	-3.0605	8.3426	5.1816	13.4320	6.5748	10.5857
HuH7 x	miR 502	15.1937	0.1112	-10.2318	0.3517	-4.4644	22.0757	13.5571	35.9468	17.2998	28.1700
HuH7 x	miR 24	25.9770	0.0291	0.5515	0.3349	-1.3159	2.4896	1.5649	3.9608	1.9739	3.1402
HuH7 x	miR 210	24.9798	0.0026	-0.4457	0.3337	-2.7929	6.9303	4.3639	11.0062	5.4994	8.7336
HuH7 x	miR 200	34.4865	0.0186	9.0609	0.3342	12.4123	0.0002	0.0001	0.0003	0.0001	0.0002
HuH7 x	miR ref	25.4255	5.3384	0.0	0.0	0.0	1.0	1.0	1.0	1.0	1.0

Total RNA samples extracted from cultured cells have been tested for verifying expression changes of 8 preselected micro-RNA (miRNA, miR) targets in real-time PCR.

Sample Names: Control HLE cells (HLE K); EV-treated HLE cells (HLE x); Target Names: miRNA targets; miR ref: reference microRNA computed for global normalization; Cq Mean: average of technical replicates' quantification cycle values; Cq SD: standard deviation of technical replicates' quantification cycle values; dCq Mean: 'delta-Cq' - an average of Cq normalized to reference miRNA; dCq SD: standard deviation of Cq normalized to reference miRNA.

ddCq: 'delta-delta-Cq' - normalized to control samples respectively; RQ: Relative Quantity of the target miRNA compared to the reference sample expression level (colored yellow); RQ Min 2sd / RQ Max 2sd: error estimations at 95% confidence interval (considering +/- 2-times standard deviation); RQ Min 1sd / RQ Max 1sd: error estimations at 90% confidence interval (considering +/- 1-time standard deviation).

Table S4. Results table of TaqMan Array Card real-time PCR experiment- HuH7 Cells treated by EVs.

HuH7 x	Target Name	dd Cq	RQ
UP	**hsa-miR-502-3p-478348_mir***	**-1.85**	**3.61**
	hsa-miR-320a-478594_mir	-1.51	2.84
	**hsa-miR-423-5p-478090_mir***	**-1.49**	**2.81**
	hsa-miR-148a-3p-477814_mir	-1.04	2.06
	**hsa-miR-125b-5p-477885_mir***	**-0.79**	**1.72**
	hsa-miR-653-5p-479134_mir	-0.67	1.59
	hsa-miR-448-478105_mir	-0.59	1.50
DOWN	**hsa-miR-200a-3p-478490_mir***	**0.12**	**0.92**
	hsa-miR-380-3p-477854_mir	0.34	0.79
	hsa-miR-652-3p-478189_mir	0.50	0.71

Total RNA samples extracted from cultured cells have been tested for expression profile of 377 micro-RNA (miRNA, miR) targets in TaqMan Array Card (TAC) real-time PCR. The miRNA profile of EV-treated HuH7 cells

(HuH7 x) is compared to the control HuH7 cells' profile. Targets in the upper part of the table showing increased expression level (UP) compared to control are colored as orange; Targets in the lower part of the table showing decreased expression level (DOWN) compared to control are colored as green.

Only those miR targets listed in this table which show at least a 50% expression difference to control. The miR targets written in bold and labeled with (*) are selected for verification experiments. Target Name: human miRNA targets with assay IDs; ddCq: 'delta-delta-Cq' - normalized to control sample; RQ: Relative Quantity of the target miRNA compared to the control sample expression level used as reference; Analysis software: Thermo Fisher Connect, Relative Quantification module; Quantification Cycle (Cq) generation method: relative threshold cycle (CRT); Analysis criteria: amplification score (AmpScore) cutoff = 1; Cq confidence (CqConf) cutoff = 0,8; Cq cutoff = 33.

Table S5. Results table comparing micro-RNA RQ data from different experiments.

**HLE x**			
**Target Name**	**TAC RQ**	**VER RQ**	**Verified?**
miR 24	11.2045	11.3963	yes
miR 222	16.3929	7.0115	yes
miR 125	5.8523	4.2870	yes
miR 210	no difference	2.6742	NA
miR 221	1.8738	1.0663	no
miR norm	1	1	1
miR 423	0.8615	0.3336	yes
miR 502	0.4447	0.0650	yes
miR 200	0.3487	0.0472	yes
**HuH7 x**			
**Target Name**	**TAC RQ**	**VER RQ**	**Verified?**
miR 24	no difference	2.4896	NA
miR 222	no difference	5.2561	NA
miR 125	1.7231	0.8397	no
miR 210	no difference	6.9303	NA
miR 221	no difference	8.3426	NA
miR ref	1	1	1
miR 423	2.8128	40.5661	yes
miR 502	3.6075	22.0757	yes
miR 200	0.9221	0.0002	yes

Comparison table of relative quantification data of 8 preselected micro-RNA targets from two different real-time PCR experiments. The expression difference of the miR targets indicated as 'yes' in the last column have been verified.

TAC RQ: Relative Quantity (RQ) data from TaqMan Array Card (TAC) real-time PCR experiment (shown in Table S2 and S4); VER RQ: Relative Quantity (RQ) data from Verification real-time PCR experiment (shown in Table S3); HLE x: EV-treated HLE cells compared to control HLE cells; HuH7 x: EV-treated HuH7 cells compared to control HuH7 cells; NA: verification was not possible as the target expression level showed no difference on TAC

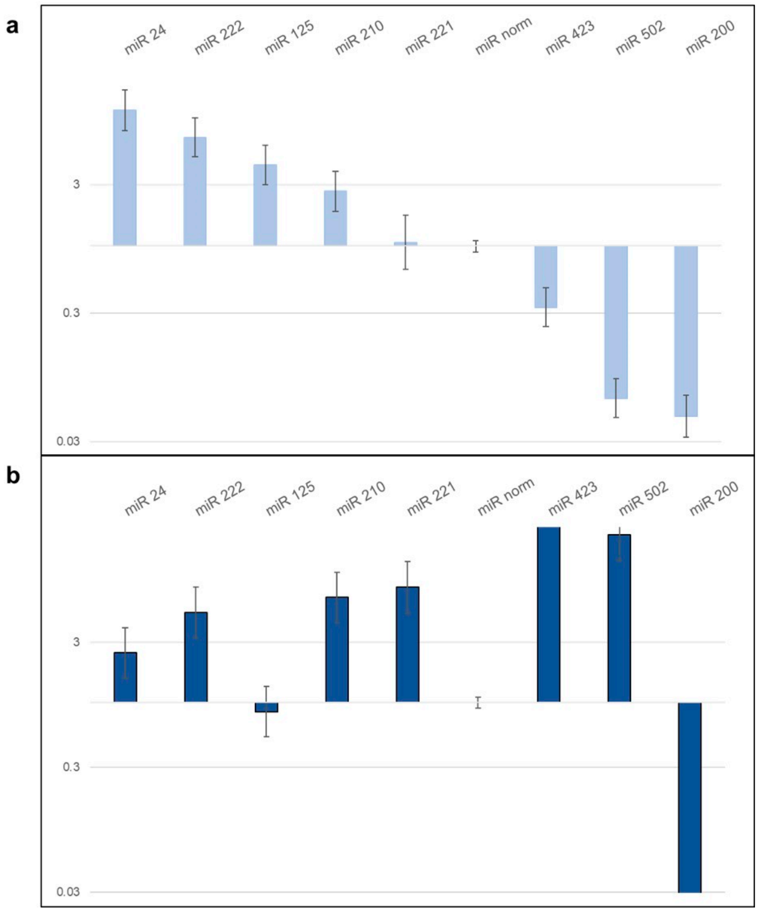

Figure S2. qRT-PCR validation of miRNAs shown to be differentially regulated by the TaqMan card in HLE (a) and HuH7 (b) upon treatment with LX2-derived EVs.

Table S6. Mean concentration values of intracellular and extracellular metabolites in HLE, HuH7, and LX2 cells, alone or in indirect co-cultures with LX2 fibroblasts or hepatoma cells. CTL: control, CM: conditioned media, CIT: citrate, LAC: lactate, MAL: malate, PYR: pyruvate

**Intracellular metabolites**	**CIT**	**LAC**	**MAL**	**PYR**
**HuH7 CTL**	815.843	3277.743	988.000	770.571
**HuH7 with LX2 CM**	955.546	11214.000	790.400	604.029
**HLE CTL**	744.136	2226.780	370.006	516.780
**HLE with LX2 CM**	924.440	6443.600	606.480	398.460
**LX2 CTL**	678.050	2566.167	467.273	739.500
**LX2 with HuH7 CM**	763.363	14744.333	764.750	1290.500
**LX2 with HLE CM**	554.537	14477.333	590.520	1357.200
**Extracellular metabolites**	**CIT**	**LAC**	**MAL**	**PYR**
**HuH7 CTL**	3624.225	211820.000	1190.350	36322.500
**HLE CTL**	5066.275	324405.000	1147.125	36257.250
**LX2 CTL**	3972.800	160867.500	924.350	44587.500

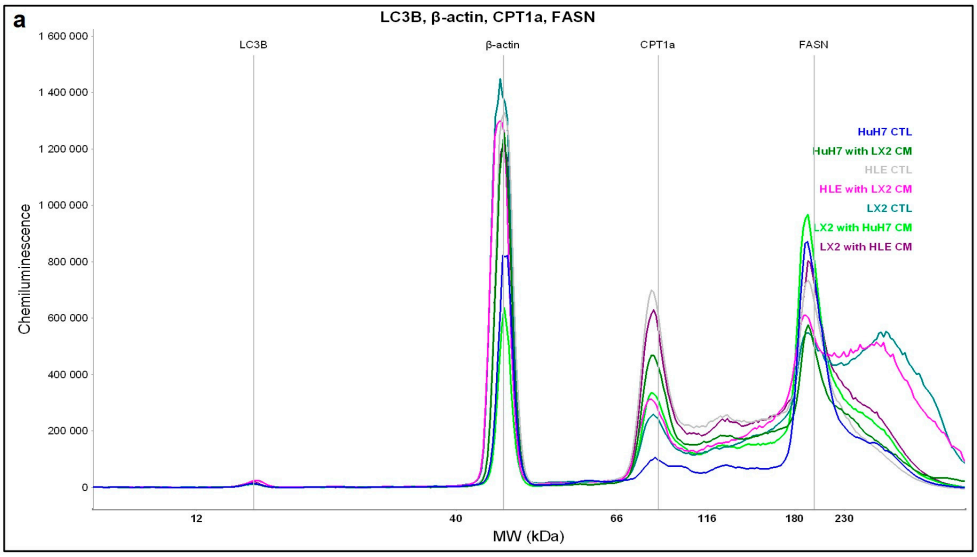
a
LC3B, β-actin, CPT1a, FASN
LC3B
β-actin
CPT1a
FASN
1 600 000
1 400 000
1 200 000
1 000 000
800 000
600 000
400 000
200 000
0
Chemiluminescence
12
40
66
116
180
230
MW (kDa)
HuH7 CTL
HuH7 with LX2 CM
HLE CTL
HLE with LX2 CM
LX2 CTL
LX2 with HuH7 CM
LX2 with HLE CM

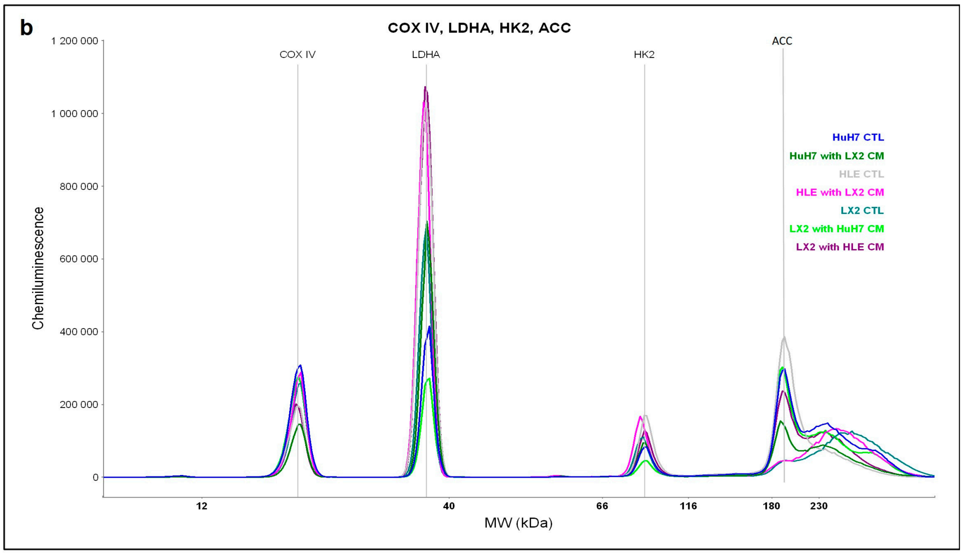
b
COX IV, LDHA, HK2, ACC
COX IV
LDHA
HK2
ACC
1 200 000
1 000 000
800 000
600 000
400 000
200 000
0
Chemiluminescence
12
40
66
116
180
230
MW (kDa)
HuH7 CTL
HuH7 with LX2 CM
HLE CTL
HLE with LX2 CM
LX2 CTL
LX2 with HuH7 CM
LX2 with HLE CM

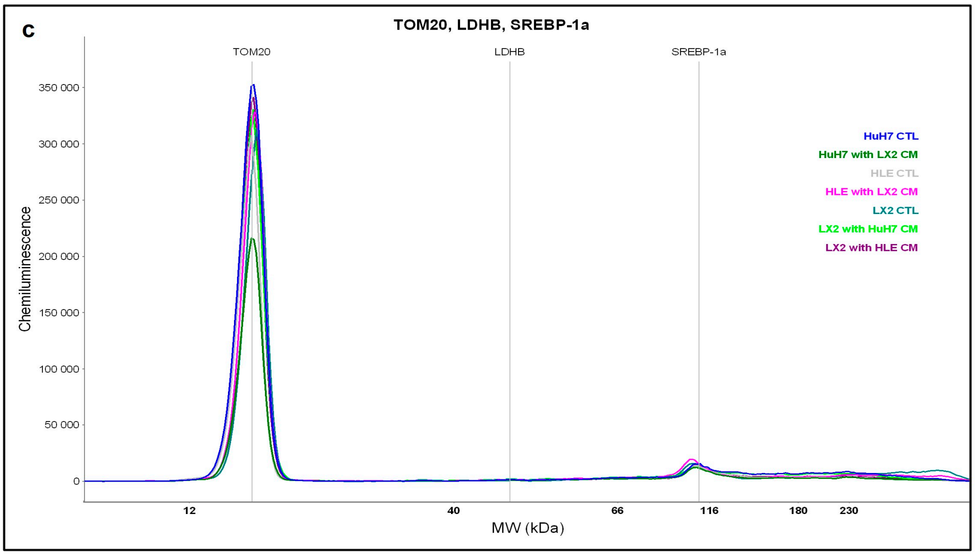
c
TOM20, LDHB, SREBP-1a
TOM20
LDHB
SREBP-1a
350 000
300 000
250 000
200 000
150 000
100 000
50 000
0
Chemiluminescence
12
40
66
116
180
230
MW (kDa)
HuH7 CTL
HuH7 with LX2 CM
HLE CTL
HLE with LX2 CM
LX2 CTL
LX2 with HuH7 CM
LX2 with HLE CM

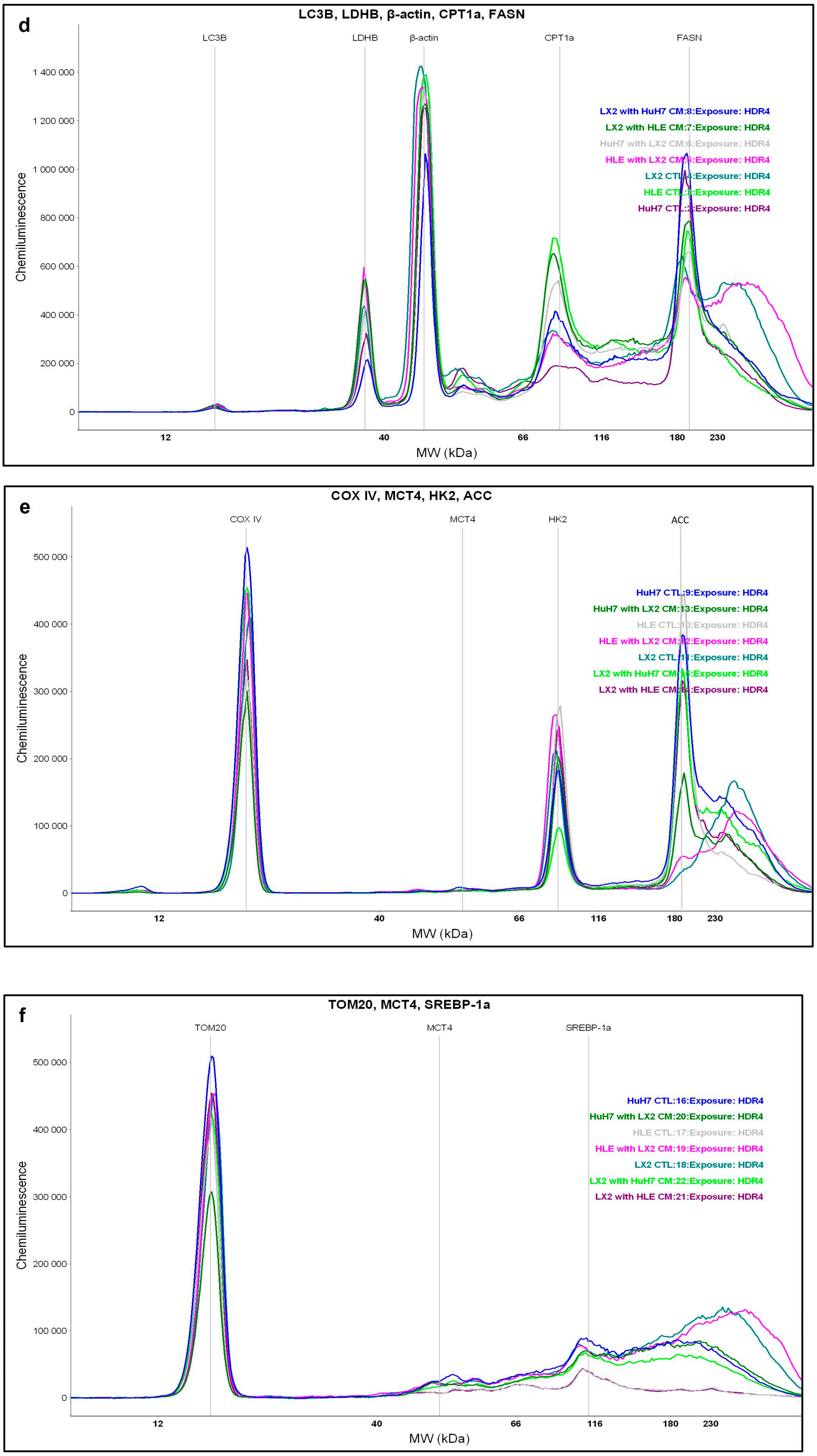

d
LC3B, LDHB, β-actin, CPT1a, FASN
LC3B
LDHB
β-actin
CPT1a
FASN
Chemiluminescence
1 400 000
1 200 000
1 000 000
800 000
600 000
400 000
200 000
0
LX2 with HuH7 CM:8:Exposure: HDR4
LX2 with HLE CM:7:Exposure: HDR4
HuH7 with LX2 CM:6:Exposure: HDR4
HLE with LX2 CM
Exposure: HDR4
LX2 CTL
Exposure: HDR4
HLE CTL
Exposure: HDR4
HuH7 CTL
Exposure: HDR4
12
40
66
116
180
230
MW (kDa)
e
COX IV, MCT4, HK2, ACC
COX IV
MCT4
HK2
ACC
Chemiluminescence
500 000
400 000
300 000
200 000
100 000
0
HuH7 CTL:9:Exposure: HDR4
HuH7 with LX2 CM:13:Exposure: HDR4
HLE CTL
Exposure: HDR4
HLE with LX2 CM
Exposure: HDR4
LX2 CTL
Exposure: HDR4
LX2 with HuH7 CM
Exposure: HDR4
LX2 with HLE CM
Exposure: HDR4
12
40
66
116
180
230
MW (kDa)
f
TOM20, MCT4, SREBP-1a
TOM20
MCT4
SREBP-1a
Chemiluminescence
500 000
400 000
300 000
200 000
100 000
0
HuH7 CTL:16:Exposure: HDR4
HuH7 with LX2 CM:20:Exposure: HDR4
HLE CTL:17:Exposure: HDR4
HLE with LX2 CM:19:Exposure: HDR4
LX2 CTL:18:Exposure: HDR4
LX2 with HuH7 CM:22:Exposure: HDR4
LX2 with HLE CM:21:Exposure: HDR4
12
40
66
116
180
230
MW (kDa)

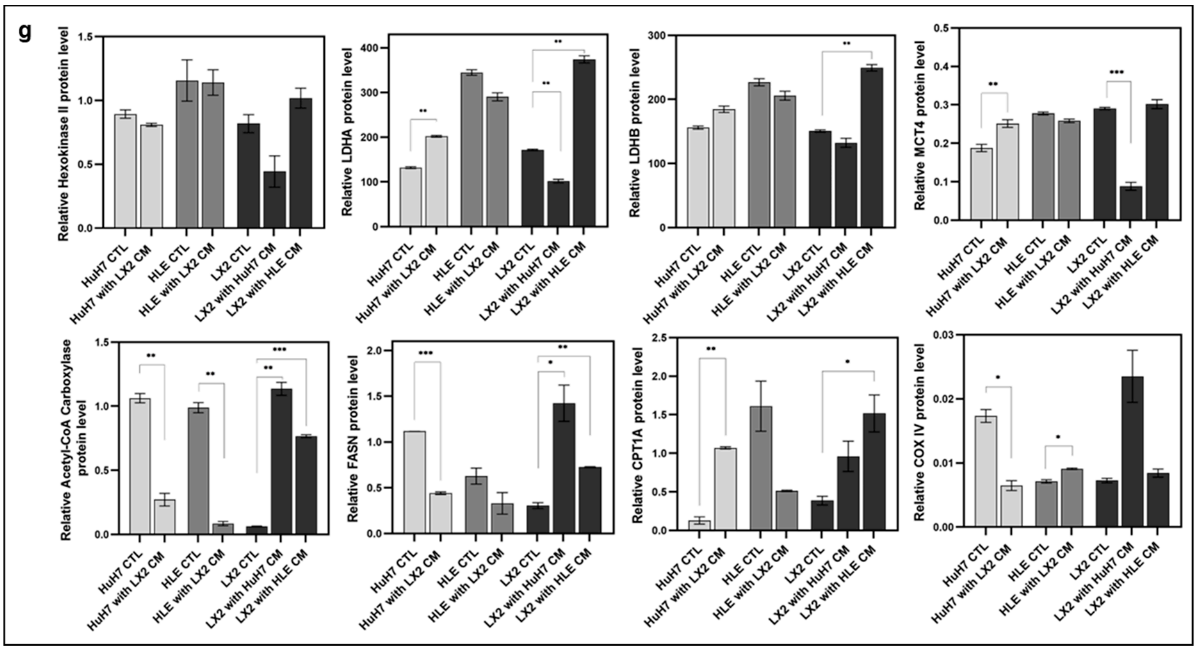

h

	CPT1A	FASN	COX IV	LDHA	LDHB	HK2	ACC	MCT4
**Huh7 CTL**	0.1291	1.1169	0.0173	132.1724	156.0655	0.8948	1.0631	0.1877
**Huh7 with LX2 CM**	1.0695	0.4401	0.0065	202.1523	184.6477	0.8094	0.2714	0.2515
**HLE CTL**	1.6120	0.6268	0.0071	344.8875	226.9885	1.1575	0.9885	0.2776
**HLE with LX2 CM**	0.5127	0.3296	0.0091	290.4200	205.7939	1.1413	0.0855	0.2587
**LX2 CTL**	0.3860	0.3052	0.0073	171.6647	150.5227	0.8190	0.0629	0.2904
**LX2 with Huh7 CM**	0.9611	1.4229	0.0235	101.9629	132.4391	0.4441	1.1363	0.0882
**LX2 with HLE CM**	1.5177	0.7255	0.0084	374.2984	249.4552	1.0195	0.7668	0.3019

Figure S3. Raw data of Figure 13. (a-f) Raw Wes simple Electropherograms, (g) corresponding densitometry graphs and (h) mean values of relative densitometry. β-actin was used as reference for protein loading and relative activity levels were compared to respective controls. Data points represent mean ± SD, n = 3; * $p < 0.05$; ** $p < 0.01$; *** $p < 0.001$. CTL: control, CM: conditioned medium, HK2: Hexokinase 2, LDHA: Lactate dehydrogenase A, LDHB: Lactate dehydrogenase B, MCT4: Monocarboxylate transporter 4, ACC: Acetyl-CoA Carboxylase, FASN: Fatty acid synthase, CPT1A: Carnitine palmitoyl transferase 1A, COXIV: Cytochrome c oxidase subunit IV.

Table S7. Antibodies used in the present study.

**Primary antibodies**	**Host**	**Manufacturer***	**Cat. no.**	**Dilution IF**	**Dilution WB, DB/Wes**
Acetyl-CoA Carboxylase (C83B10)	Rabbit monoclonal	Cell Signaling	3676	-	1:50
Anti-Lactate Dehydrogenase B/LDH-B	Mouse monoclonal	Abcam	85319	-	1:50
Beclin-1	Rabbit monoclonal	Cell Signaling	3495	-	1:1000
Calnexin	Rabbit monoclonal	Cell Signaling	2679	-	1:1000
CD44	Mouse monoclonal	Antibodies online	ABIN96695	-	1:500
CD63	Rabbit polyclonal	Santa Cruz	sc-15363	-	1:1000
Collagen IV	Mouse monoclonal	Dako	M 0785	-	1:200
COX IV (3E11)	Rabbit monoclonal	Cell Signaling	4850	-	1:50
CPT1A	Mouse monoclonal	Abcam	128568	-	1:50
Cytokeratin	Mouse monoclonal	Dako	M3515	1:100	-
Fatty Acid Synthase (FASN)	Rabbit monoclonal	Cell Signaling	3180	-	1:50
Fibronectin (IST-9)	Mouse monoclonal	Santa Cruz	sc-59826	-	1:500
GSK-3α/β	Rabbit monoclonal	Cell Signaling	5676	-	1:1000
Hexokinase II (C64G5)	Rabbit monoclonal	Cell Signaling	2867	-	1:50
Integrin α5	Rabbit polyclonal	Cell Signaling	4705	-	1:1000

Integrin α6	Rabbit polyclonal	Cell Signaling	3750	-	1:750
Integrin αV	Rabbit polyclonal	Cell Signaling	4711	-	1:1000
Integrin β1	Rabbit polyclonal	Cell Signaling	4706	-	1:1000
Integrin β4	Rabbit polyclonal	Cell Signaling	4707	-	1:1000
Laminin β1 (H-300)	Rabbit polyclonal	Santa Cruz	sc-13587	-	1:200
LC3	Rabbit polyclonal	Cell Signaling	2775	-	1:1000/ 1:50
LDHA (C4B5)	Rabbit monoclonal	Cell Signaling	3582	-	1:50
MCT4	Rabbit polyclonal	Genetex	GTX131626	-	1:50
NF-kB p65 (D14E12)	Rabbit monoclonal	Cell Signaling	8242	-	1:1000
p44/42	Rabbit monoclonal	Cell Signaling	4695	-	1:1000
pAKT (S473)	Rabbit monoclonal	Cell Signaling	4058	-	1:1000
pAkt (T308)	Rabbit monoclonal	Cell Signaling	2965	-	1:1000
pGSK-3α/β (S21/9)	Rabbit monoclonal	Cell Signaling	8566	-	1:1000
p-p44/42 (Erk1/2) (T202/Y204)	Rabbit monoclonal	Cell Signaling	4370	-	1:1000
SREBP-1a	Rabbit polyclonal	Invitrogen	PA13-37	-	1:50
Syndecan-1 (SDC1)	Mouse monoclonal	Dako	M7228	-	1:1000
Testican-1	Rabbit polyclonal	Atlas Antibodies	HPA07450	1:200	-
Thrombospondin 1 (A6.1)	Mouse monoclonal	Santa Cruz	sc-59887	-	1:250
Tom20	Rabbit monoclonal	Cell Signaling	42406	-	1:500/ 1:50
Vimentin	Mouse monoclonal	Dako	m0725	1:100	-
α-SMA (Smooth Muscle Actin)	Mouse monoclonal	Dako	M0851	1:200	-
β-actin	Mouse monoclonal	Sigma Aldrich	A2228	-	1:5000
β-actin	Rabbit polyclonal	Cell Signaling	4967	-	1:1000
β-catenin	Rabbit polyclonal	Cell Signaling	9562	-	1:1000
**Secondary antibody**	**Host**	**Manufacturer***	**Cat. no.**	**Dilution IF**	**Dilution WB,**
Alexa Fluor® 488 anti-rabbit IgG	Goat polyclonal	Invitrogen	a-11070	1:200	-
Alexa Fluor® 568 anti-mouse IgG	Goat polyclonal	Invitrogen	a-11004	1:200	-
Alexa Fluor® 488 anti-mouse IgG	Goat polyclonal	Invitrogen	a-11029	1:200	-
Anti-Rabbit immunglobulin/HRP	Goat Polyclonal	Dako	P 0448	-	1:2000
Anti-Mouse Immunglobulin/HRP	Goat Polyclonal	Dako	P 0447	-	1:2000

* Abcam, Cambridge, UK; Atlas Antibodies, Stockholm, Sweden, Cell Signaling Technology, Danvers, MA, USA; Dako/DakoCytomation; Genetex, CA, USA, Invitrogen by Life Technologies, Carlsbad, CA, USA; Santa Cruz Biotechnology, CA, USA, Sigma-Aldrich Co., St. Louis, MO, USA, IF: Immunofluorescence staining, WB: Western blot, DB: Dot Blot

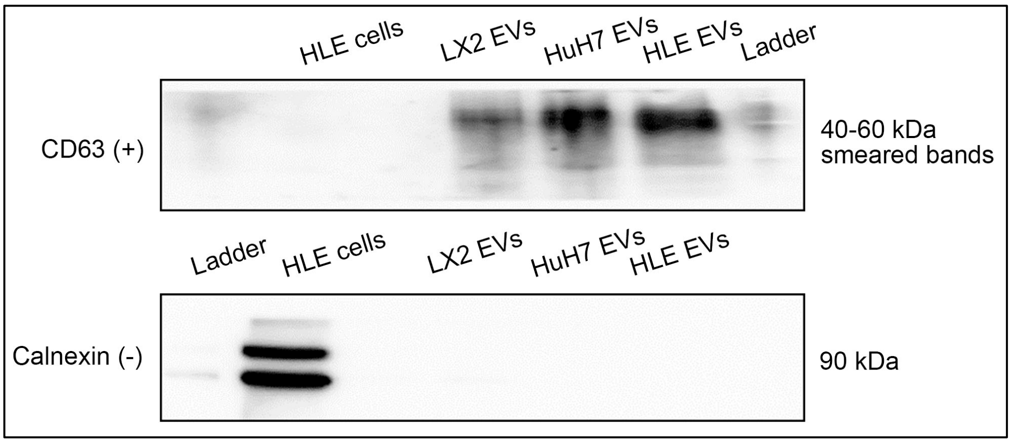

Figure S4. The purity of the EV isolates were determined using positive (CD63) and negative (calnexin) markers with Western Blot. EV extracts derived from HLE, HuH7, and LX2 were analyzed for the presence of protein CD63. HLE cell lysates were used as a control. The results indicated the presence of CD63 in the isolated EVs, while calnexin, a negative marker, was only detected in the cell lysates.

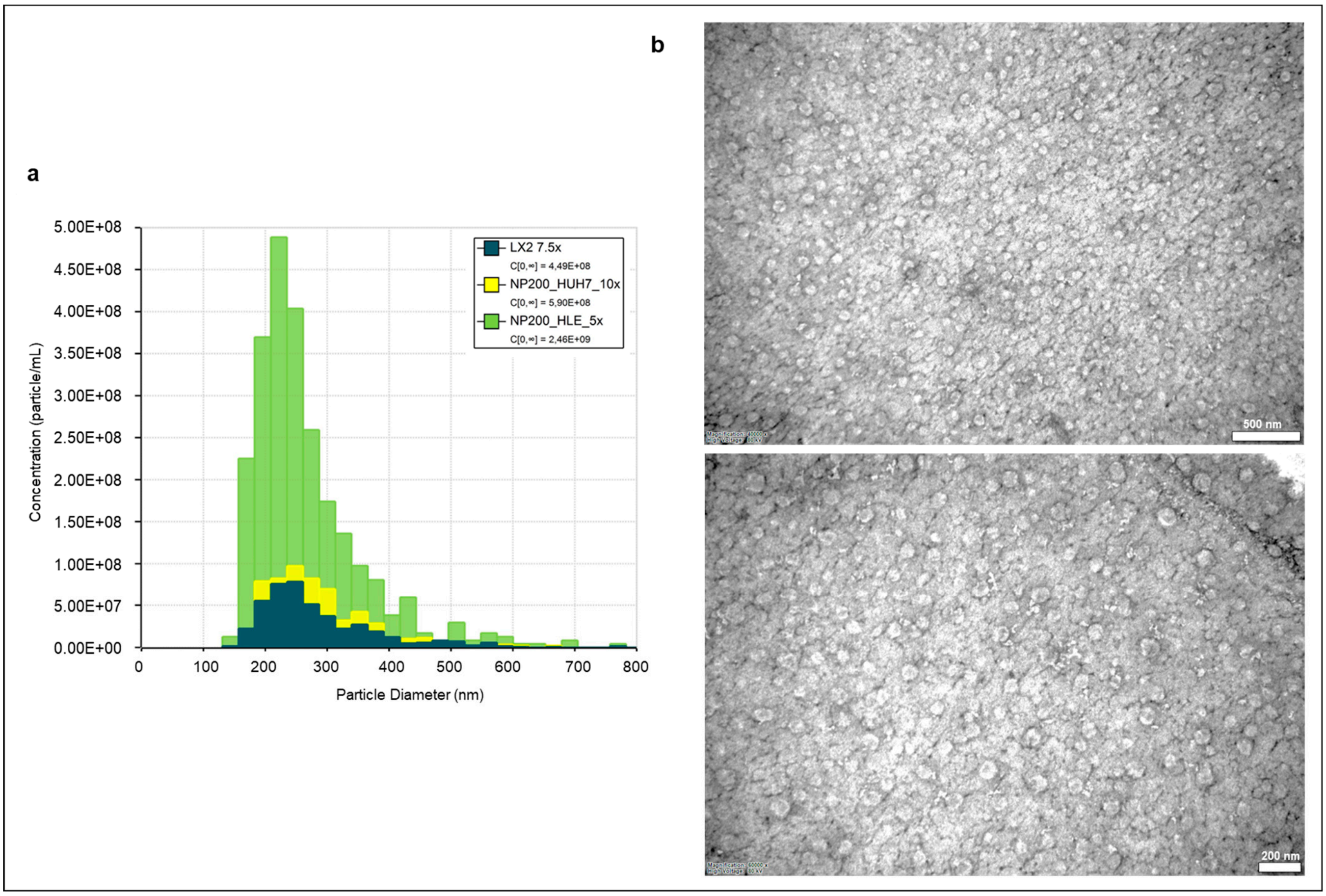

Figure S5. Characterization of EVs released by hepatoma cells (HLE, HuH7) and LX2 stellate cells. (a) Concentration values of EV particles were determined by tunable resistive pulse sensing (TRPS). Histogram plots of EVs are representative of three independent experiments. (b) Transmission electron microscopy images of HLE EVs.

Supplementary Material and methods

Size distribution and concentration of the EVs

EVs were analyzed using tunable resistive pulse sensing (TRPS) with a qNano instrument from IZON Science in Cambridge, MA, USA. The EV fraction was serially diluted in 0.2 µm filtered PBS and measured using qNano. The measurement involved particle counting for at least 3 minutes, with 5 mbar pressure and the use of NP200 nanopore membranes stretched between 45 and 47 mm. A voltage ranging from 0.1 to 0.4 V was applied to achieve a stable 120 nA current. Particle size histograms were recorded when the root mean square noise was below 12 pA, the particle rate in time was linear, and at least 500 events were counted. The calibration was conducted using a known concentration of beads (CPC100B, CPC800D, and CPC1000E) from IZON, which were diluted 1:1 000 in 0.2 µm filtered PBS and had mode diameters of 110 nm, 740 nm, and 900 nm, respectively. The results were analyzed using IZON Control Suite 3.2 software.

Transmission Electron Microscopy

Pelleted EVs were resuspended and washed in 1x sterile PBS and centrifuged at 100 000 × g at 4°C for 1 hour followed by fixation in 2.5% glutaraldehyde for 1 hour at 4°C. The glutaraldehyde-fixed samples were subjected to three washes in 0.1 M cacodylate buffer. To prepare for araldite embedding, fixed samples were dehydrated in an ascending ethanol series, followed by treatment with 1% uranyl acetate in 70% ethanol at 4 °C for 1 hour. For contrast staining, uranyl acetate, and lead (II) nitrate were applied to the ultrathin sections. The samples were examined using a Hitachi H-7600 (Tokyo, Japan) transmission electron microscope.
